# Supplementary material for: Timing the evolution of phosphorus-cycling enzymes through geological time using phylogenomics
Source: Nat Commun. 2024 May 2;15:3703. doi: 10.1038/s41467-024-47914-0 (PMC11066067; doi:10.1038/s41467-024-47914-0)
Supplement: Supplementary file 1 — Supplementary Information [file 41467_2024_47914_MOESM1_ESM.pdf]

Timing the Evolution of Phosphorus-Cycling Enzymes Through Geological Time using Phylogenomics

SUPPLEMENTARY INFORMATION

SUPPLEMENTARY DISCUSSION

Discussion of HGT Costs

The default costs for gene transfers, duplications, losses and speciations have been found to minimise genome size variation between parent and daughter lineages [1], so if HGT costs are cheaper or more expensive, we would expect to see more genome size variation over time. Whether genome size would have remained constant between parent and daughter lineages on timescales of the order of hundreds of millions of years can be debated. Some findings suggest that genome size distributions are strongly linked to evolutionary history [2] implying little change across lineages. Whereas others have found that terrestrial prokaryotes have larger genomes than aquatic and host-associated prokaryotes, implying that related organisms from different habitats would have different genome sizes [3]. For these reasons, we present the findings of reconciliations made with a variety of costs in the main paper.

SUPPLEMENTARY FIGURES

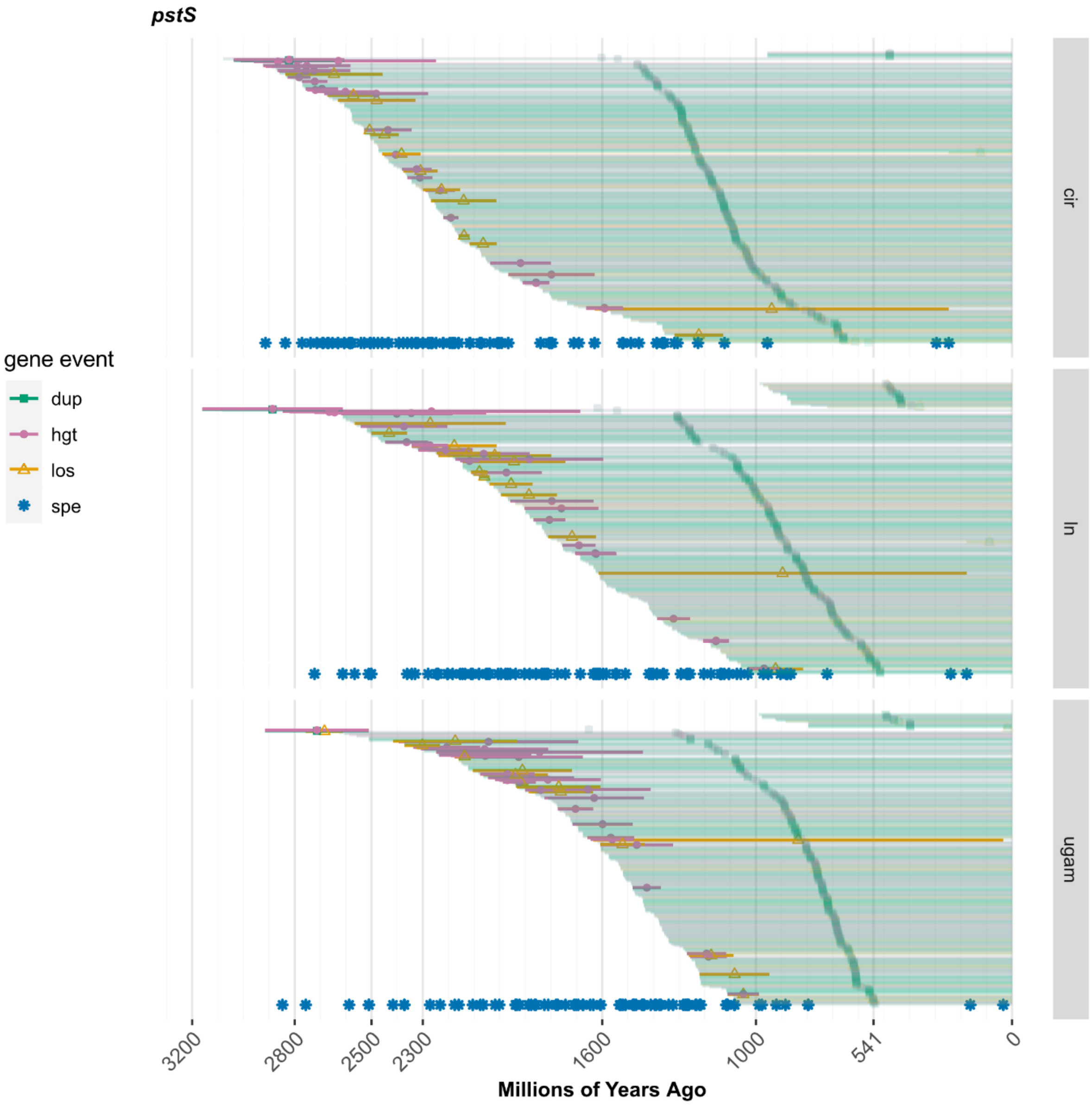

Figure 1: Uncertainty in estimating the origin of *pstS*. Horizontal lines represent the lengths of branches where gene duplications (green), horizontal gene transfers (pink) and losses (orange) are predicted to have occurred. The midpoint of each branch is marked with shapes of the same colour, representing duplications (filled squares), horizontal gene transfers (filled circles) and losses (empty triangles). Darkness indicates whether the event occurred on an internal (dark colour) or terminal (faded) branch of the tree of life. Gene speciations (blue asterisks) are not associated with branch lengths because they occur on internal nodes of the tree. Results found using three different clock models are shown (cir: Cox-Ingersoll-Ross, ln: lognormal, ugam: uncorrelated gamma multipliers).

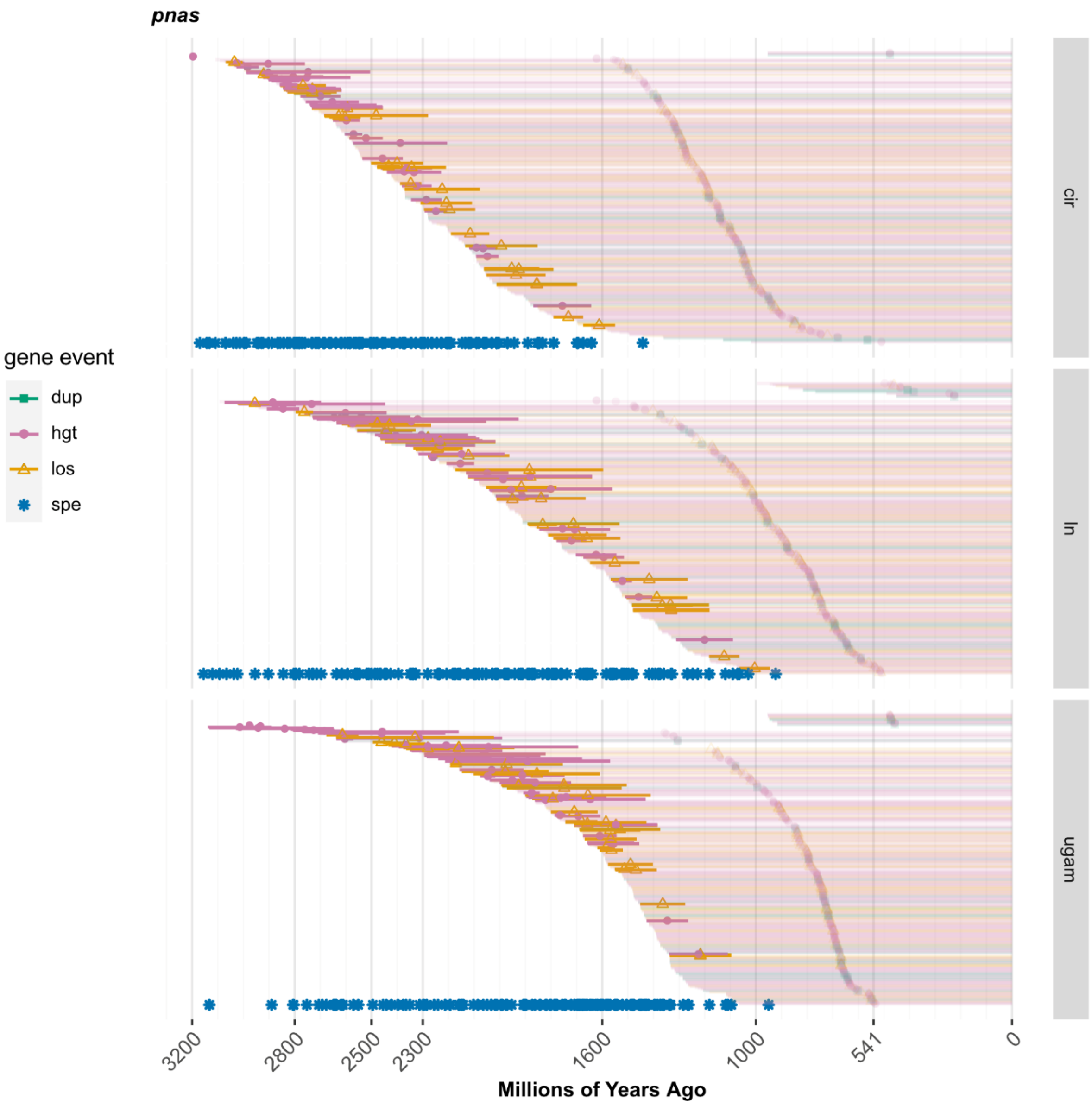

Figure 2: Uncertainty in estimating the origin of PNaS. Horizontal lines represent the lengths of branches where gene duplications (green), horizontal gene transfers (pink) and losses (orange) are predicted to have occurred. The midpoint of each branch is marked with shapes of the same colour, representing duplications (filled squares), horizontal gene transfers (filled circles) and losses (empty triangles). Darkness indicates whether the event occurred on an internal (dark colour) or terminal (faded) branch of the tree of life. Gene speciations (blue asterisks) are not associated with branch lengths because they occur on internal nodes of the tree. Results found using three different clock models are shown (cir: Cox-Ingersoll-Ross, ln: lognormal, ugam: uncorrelated gamma multipliers).

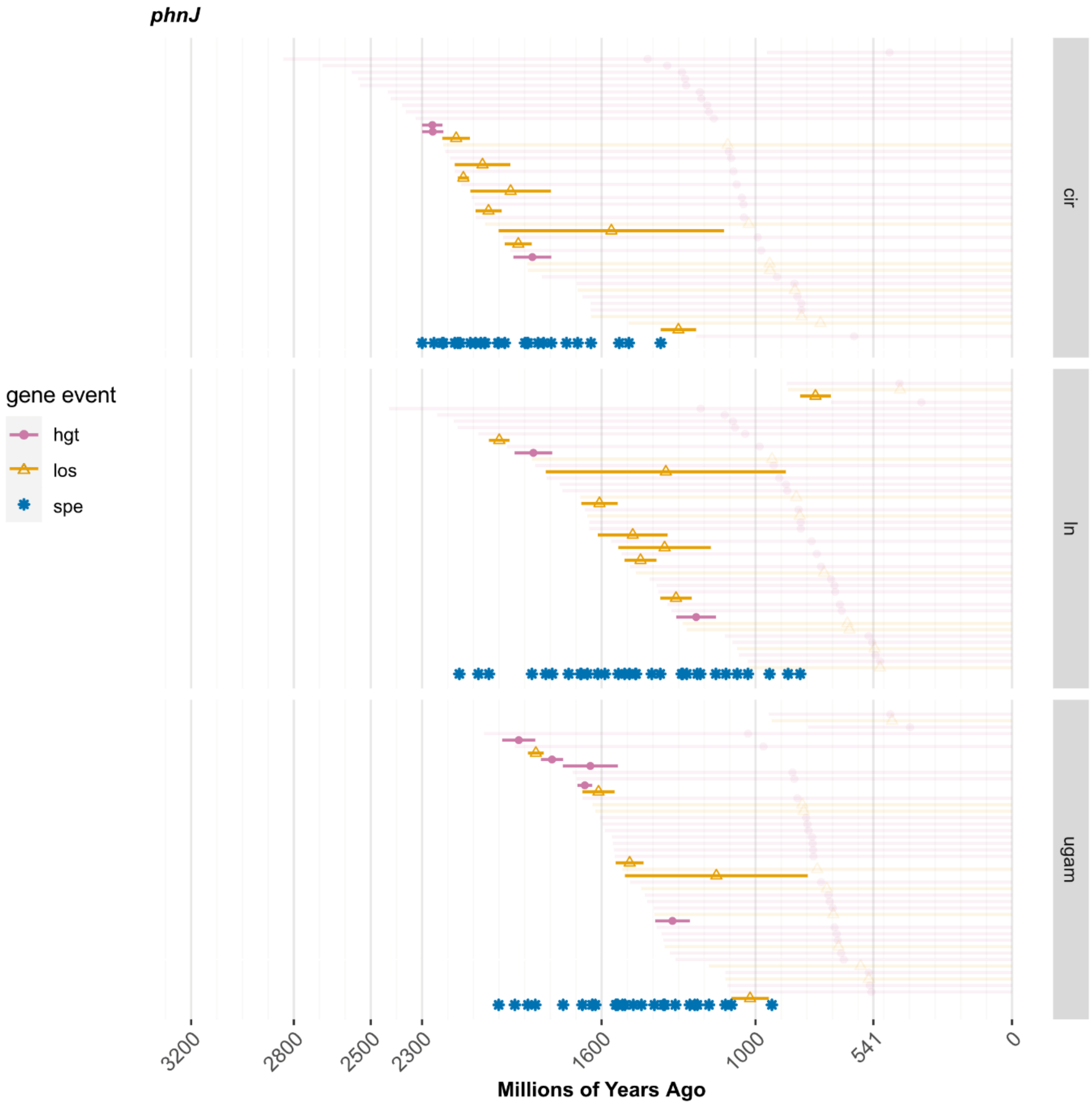

Figure 3: Uncertainty in estimating the origin of *phnJ*. Horizontal lines represent the lengths of branches where gene duplications (green), horizontal gene transfers (pink) and losses (orange) are predicted to have occurred. The midpoint of each branch is marked with shapes of the same colour, representing duplications (filled squares), horizontal gene transfers (filled circles) and losses (empty triangles). Darkness indicates whether the event occurred on an internal (dark colour) or terminal (faded) branch of the tree of life. Gene speciations (blue asterisks) are not associated with branch lengths because they occur on internal nodes of the tree. Results found using three different clock models are shown (cir: Cox-Ingersoll-Ross, ln: lognormal, ugam: uncorrelated gamma multipliers).

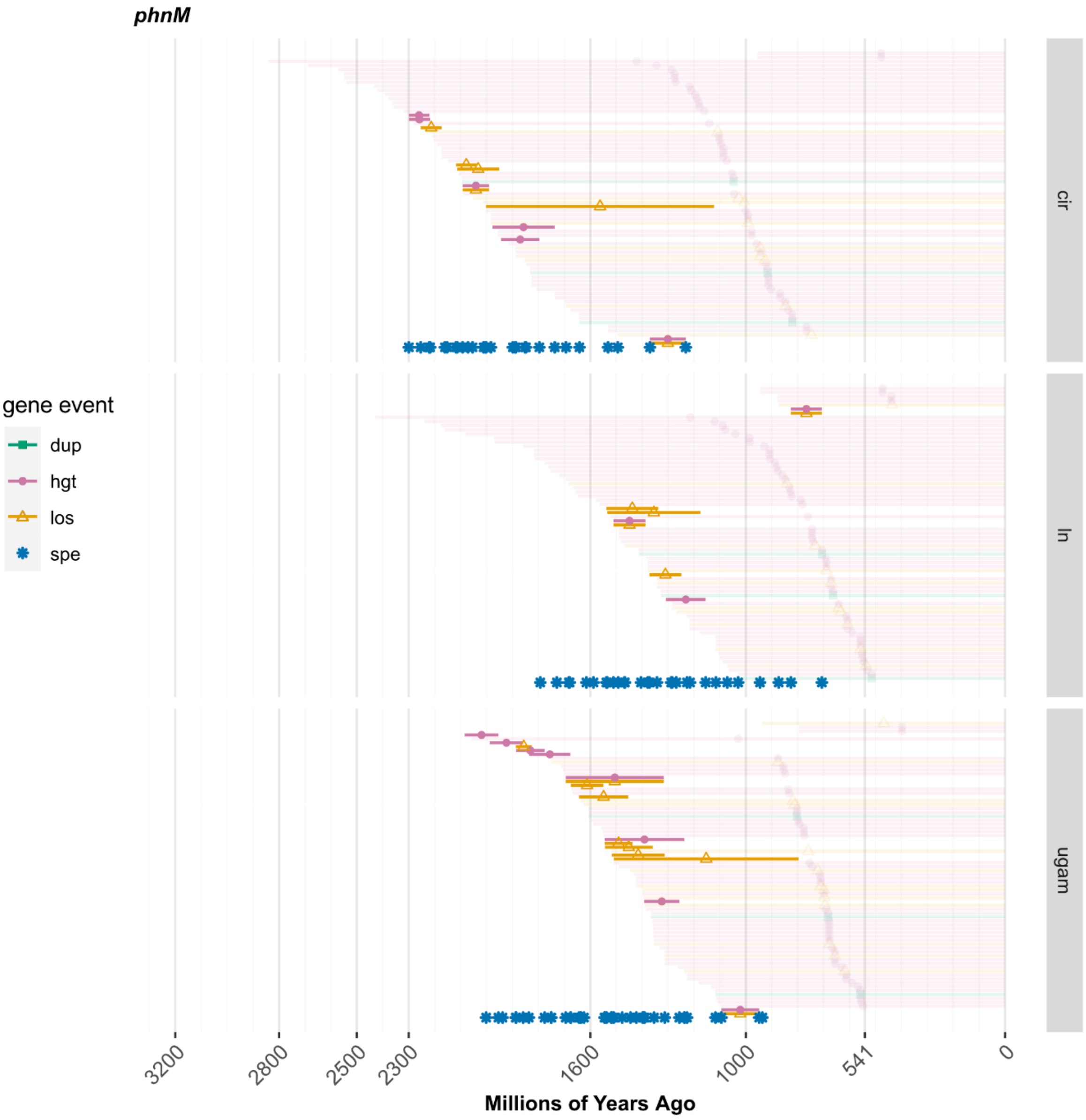

Figure 4: Uncertainty in estimating the origin of *phnM*. Horizontal lines represent the lengths of branches where gene duplications (green), horizontal gene transfers (pink) and losses (orange) are predicted to have occurred. The midpoint of each branch is marked with shapes of the same colour, representing duplications (filled squares), horizontal gene transfers (filled circles) and losses (empty triangles). Darkness indicates whether the event occurred on an internal (dark colour) or terminal (faded) branch of the tree of life. Gene speciations (blue asterisks) are not associated with branch lengths because they occur on internal nodes of the tree. Results found using three different clock models are shown (cir: Cox-Ingersoll-Ross, ln: lognormal, ugam: uncorrelated gamma multipliers).

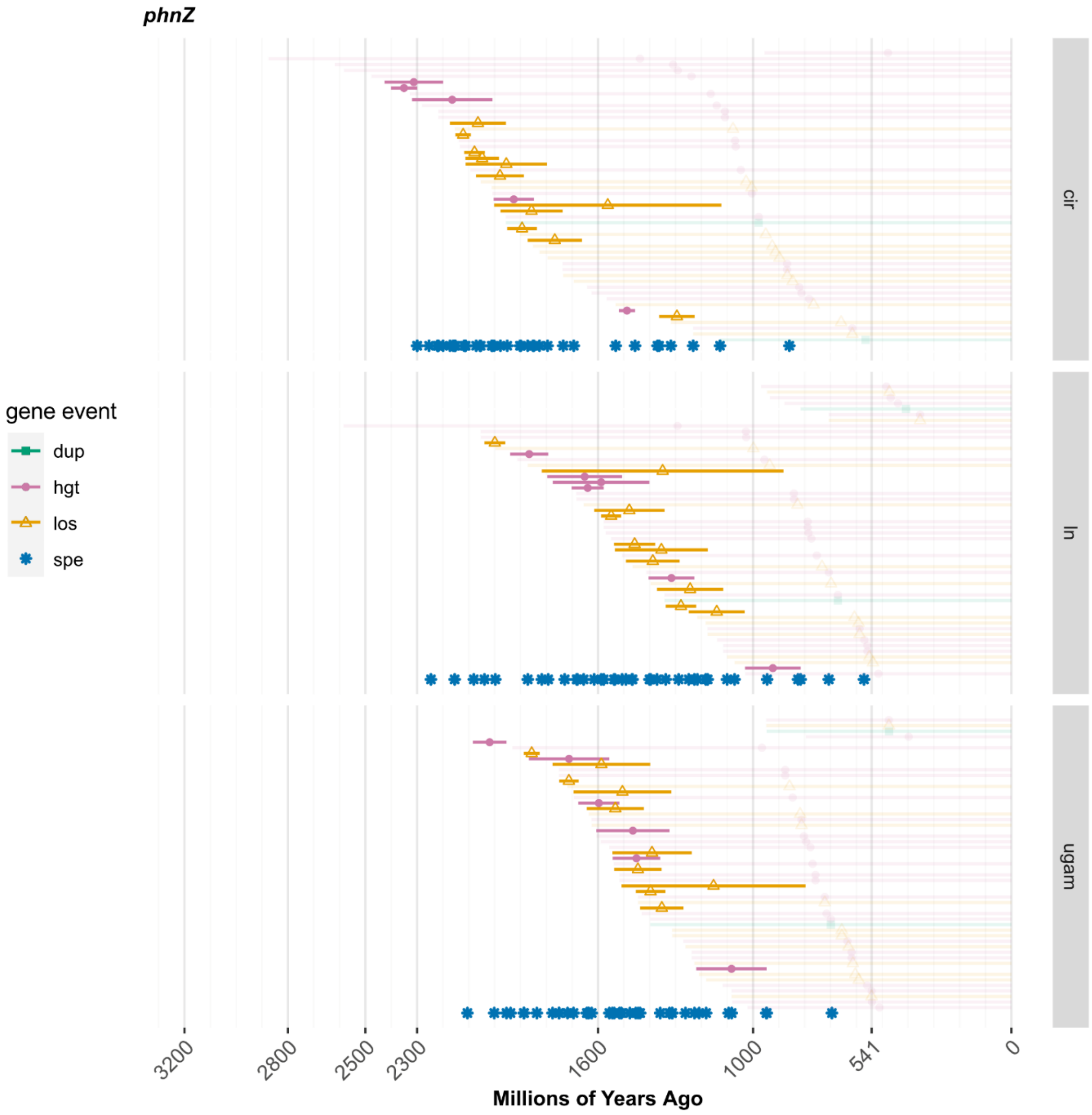

Figure 5: Uncertainty in estimating the origin of *phnZ*. Horizontal lines represent the lengths of branches where gene duplications (green), horizontal gene transfers (pink) and losses (orange) are predicted to have occurred. The midpoint of each branch is marked with shapes of the same colour, representing duplications (filled squares), horizontal gene transfers (filled circles) and losses (empty triangles). Darkness indicates whether the event occurred on an internal (dark colour) or terminal (faded) branch of the tree of life. Gene speciations (blue asterisks) are not associated with branch lengths because they occur on internal nodes of the tree. Results found using three different clock models are shown (cir: Cox-Ingersoll-Ross, ln: lognormal, ugam: uncorrelated gamma multipliers).

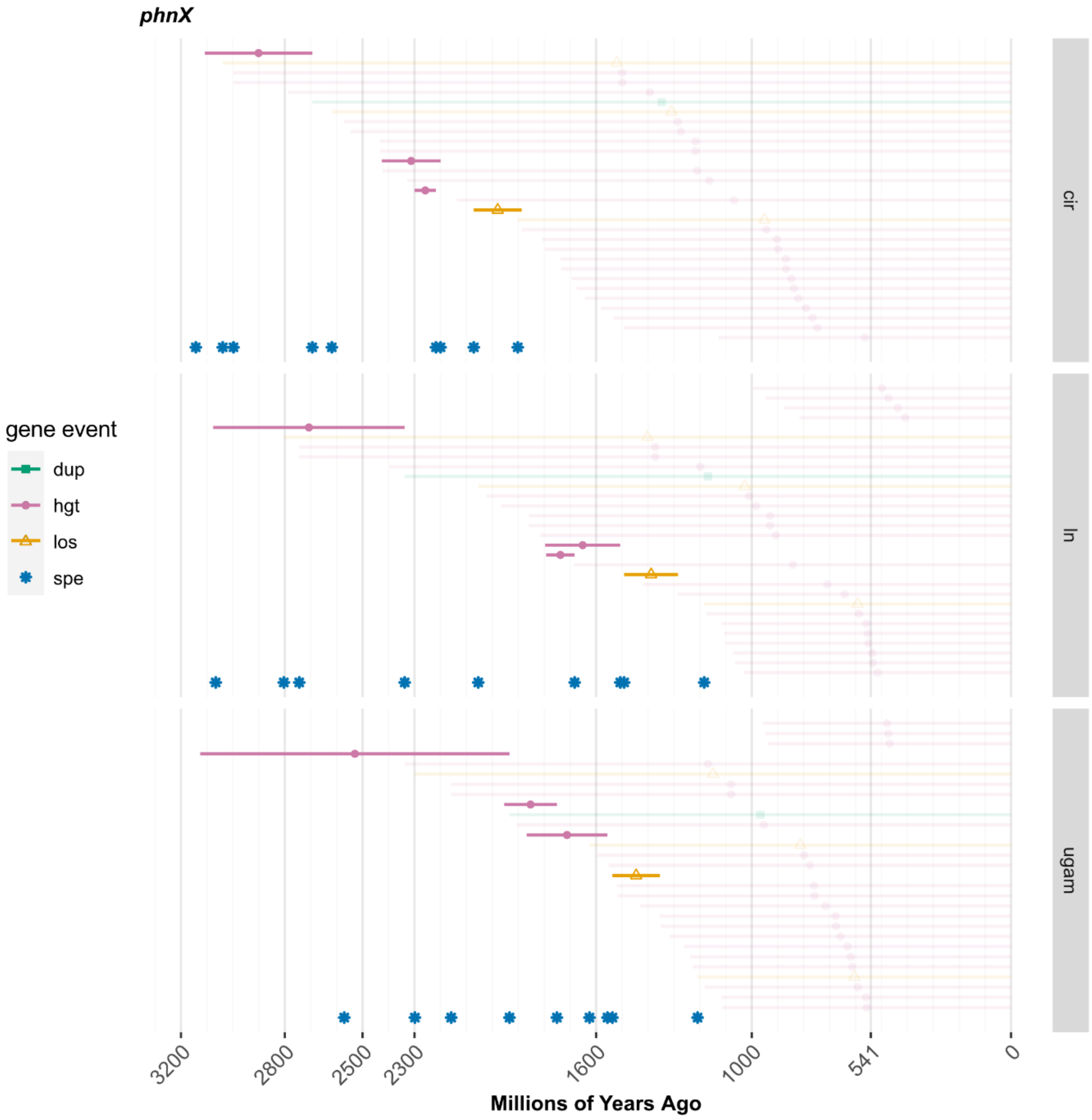

Figure 6: Uncertainty in estimating the origin of *phnX*. Horizontal lines represent the lengths of branches where gene duplications (green), horizontal gene transfers (pink) and losses (orange) are predicted to have occurred. The midpoint of each branch is marked with shapes of the same colour, representing duplications (filled squares), horizontal gene transfers (filled circles) and losses (empty triangles). Darkness indicates whether the event occurred on an internal (dark colour) or terminal (faded) branch of the tree of life. Gene speciations (blue asterisks) are not associated with branch lengths because they occur on internal nodes of the tree. Results found using three different clock models are shown (cir: Cox-Ingersoll-Ross, ln: lognormal, ugam: uncorrelated gamma multipliers).

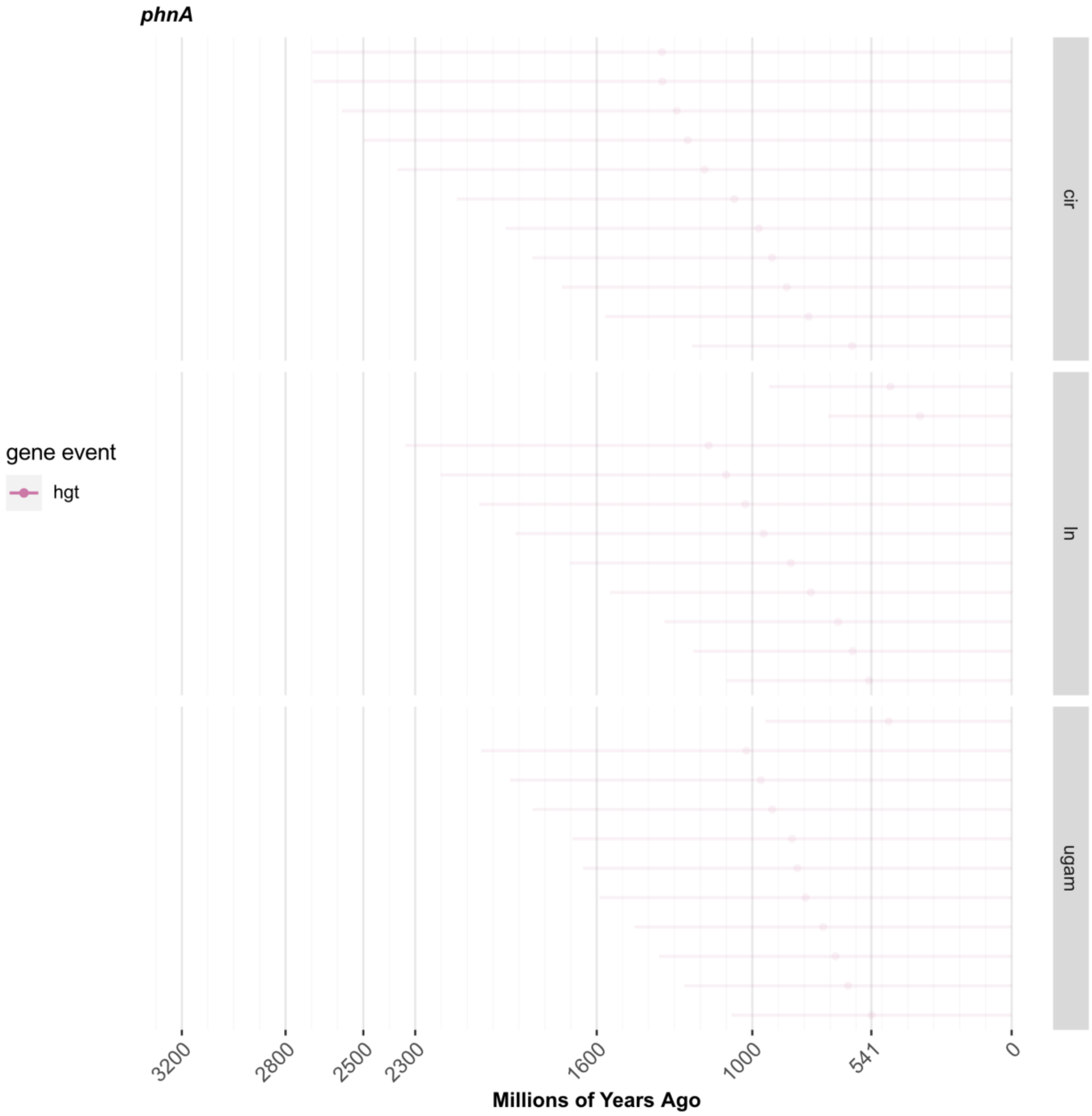

Figure 7: Uncertainty in estimating the origin of *phnA*. Horizontal lines represent the lengths of branches where horizontal gene transfers (pink) are predicted to have occurred on internal branches of the tree of life. The midpoint of each branch is marked with filled circles of the same colour. Results found using three different clock models are shown (cir: Cox-Ingersoll-Ross, ln: lognormal, ugam: uncorrelated gamma multipliers).

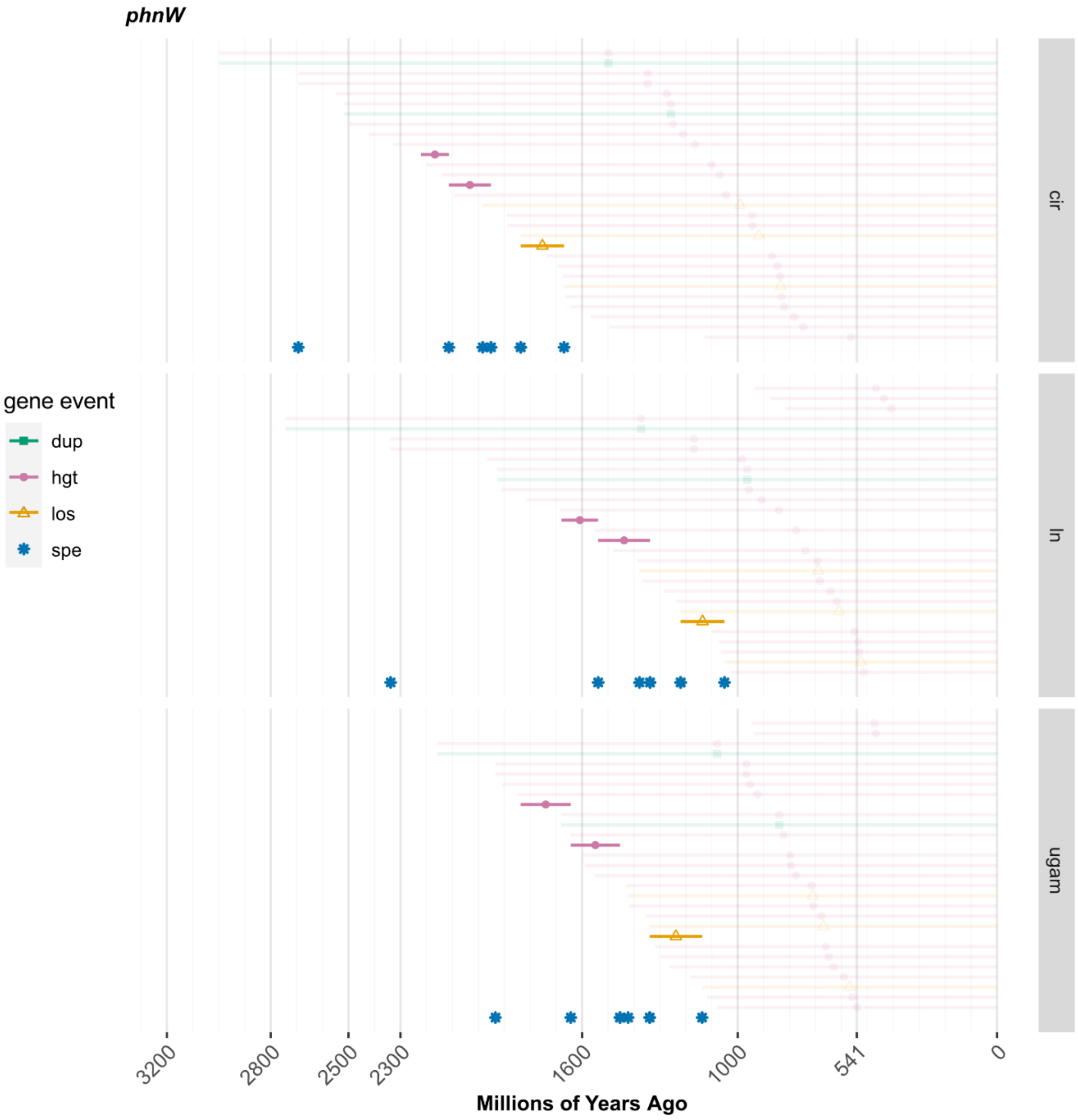

Figure 8: Uncertainty in estimating the origin of *phnW*. Horizontal lines represent the lengths of branches where gene duplications (green), horizontal gene transfers (pink) and losses (orange) are predicted to have occurred. The midpoint of each branch is marked with shapes of the same colour, representing duplications (filled squares), horizontal gene transfers (filled circles) and losses (empty triangles). Darkness indicates whether the event occurred on an internal (dark colour) or terminal (faded) branch of the tree of life. Gene speciations (blue asterisks) are not associated with branch lengths because they occur on internal nodes of the tree. Results found using three different clock models are shown (cir: Cox-Ingersoll-Ross, ln: lognormal, ugam: uncorrelated gamma multipliers).

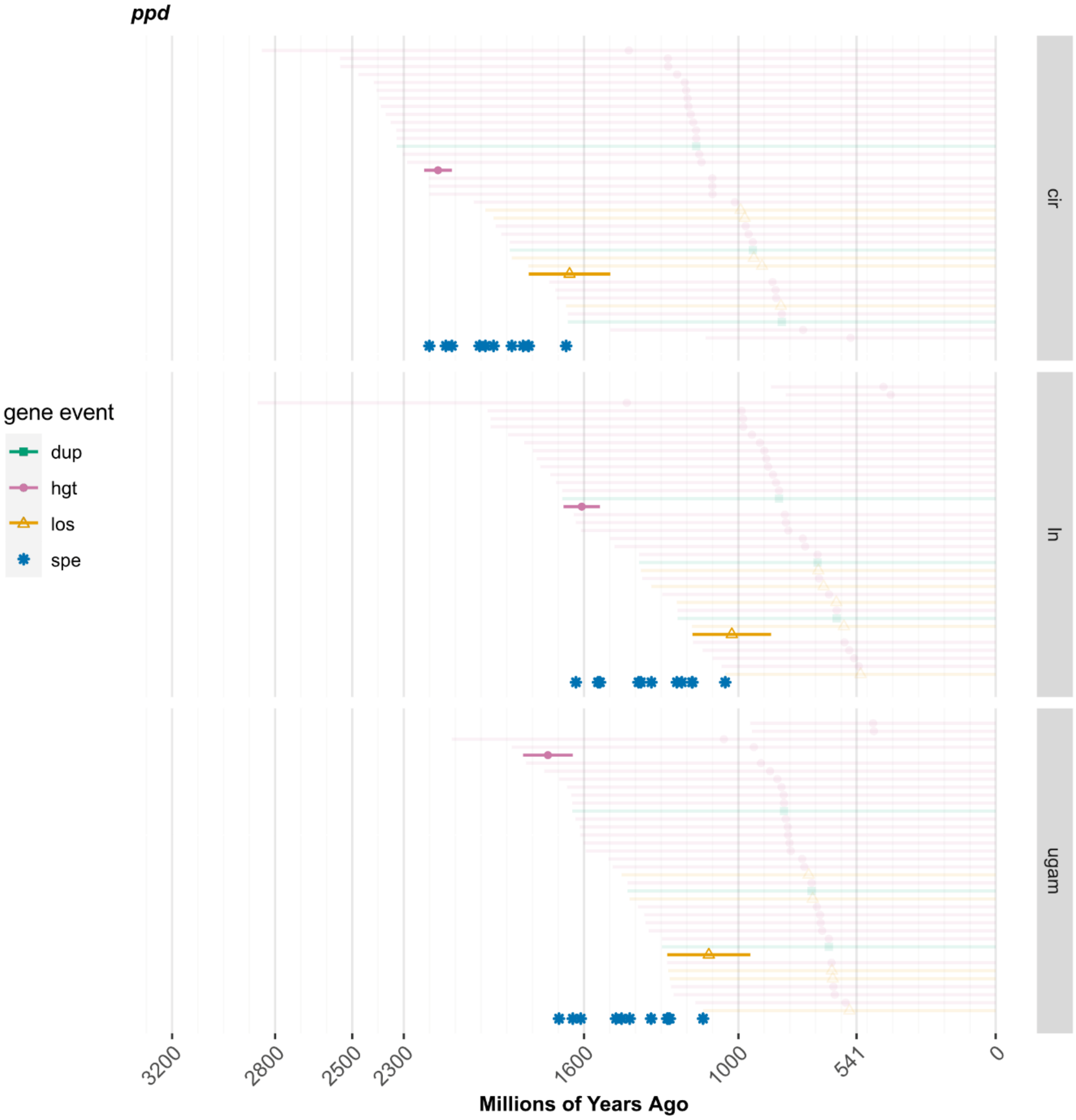

Figure 9: Uncertainty in estimating the origin of *ppd*. Horizontal lines represent the lengths of branches where gene duplications (green), horizontal gene transfers (pink) and losses (orange) are predicted to have occurred. The midpoint of each branch is marked with shapes of the same colour, representing duplications (filled squares), horizontal gene transfers (filled circles) and losses (empty triangles). Darkness indicates whether the event occurred on an internal (dark colour) or terminal (faded) branch of the tree of life. Gene speciations (blue asterisks) are not associated with branch lengths because they occur on internal nodes of the tree. Results found using three different clock models are shown (cir: Cox-Ingersoll-Ross, ln: lognormal, ugam: uncorrelated gamma multipliers).

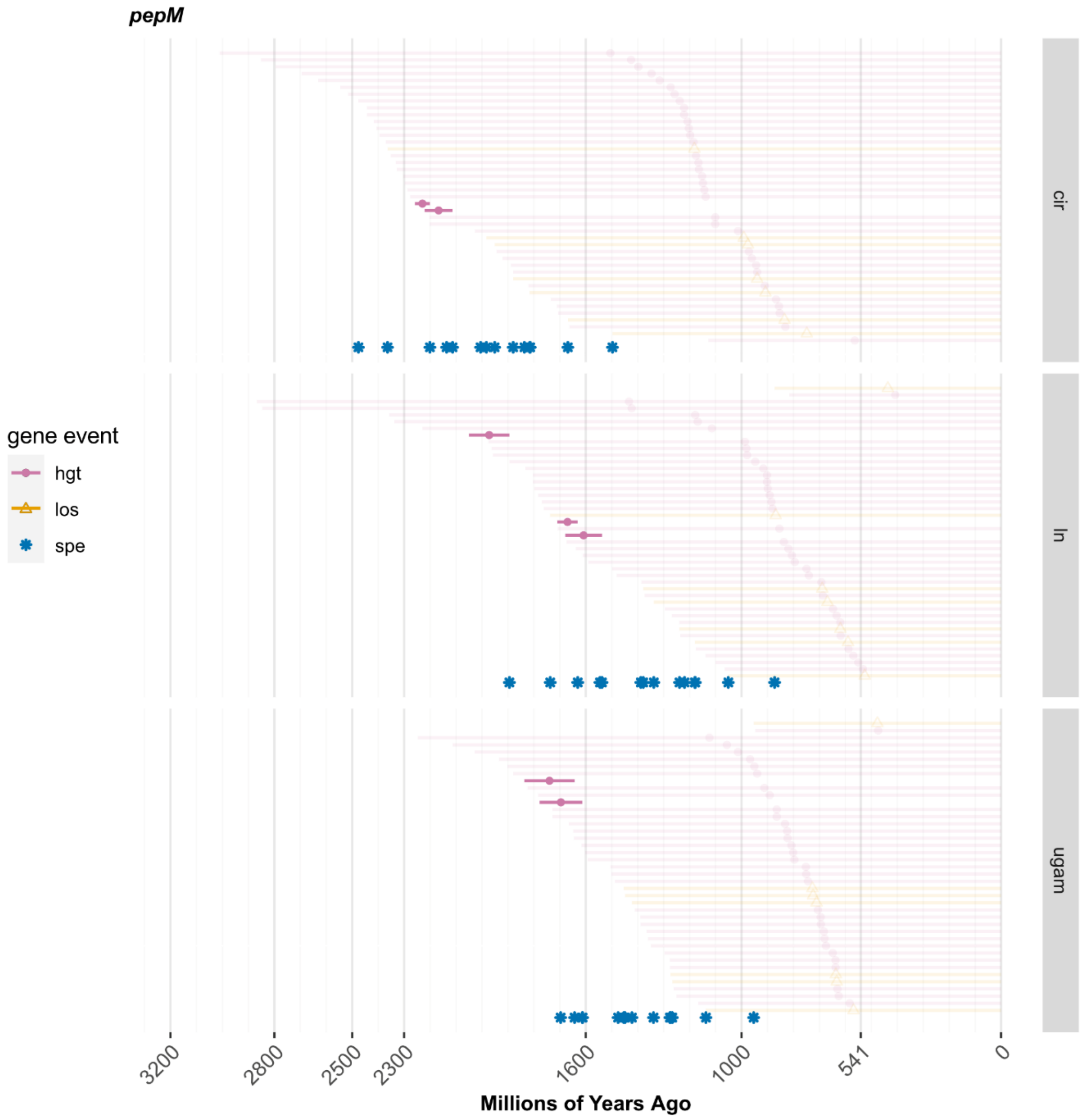

Figure 10: Uncertainty in estimating the origin of *pepM*. Horizontal lines represent the lengths of branches where gene duplications (green), horizontal gene transfers (pink) and losses (orange) are predicted to have occurred. The midpoint of each branch is marked with shapes of the same colour, representing duplications (filled squares), horizontal gene transfers (filled circles) and losses (empty triangles). Darkness indicates whether the event occurred on an internal (dark colour) or terminal (faded) branch of the tree of life. Gene speciations (blue asterisks) are not associated with branch lengths because they occur on internal nodes of the tree. Results found using three different clock models are shown (cir: Cox-Ingersoll-Ross, ln: lognormal, ugam: uncorrelated gamma multipliers).

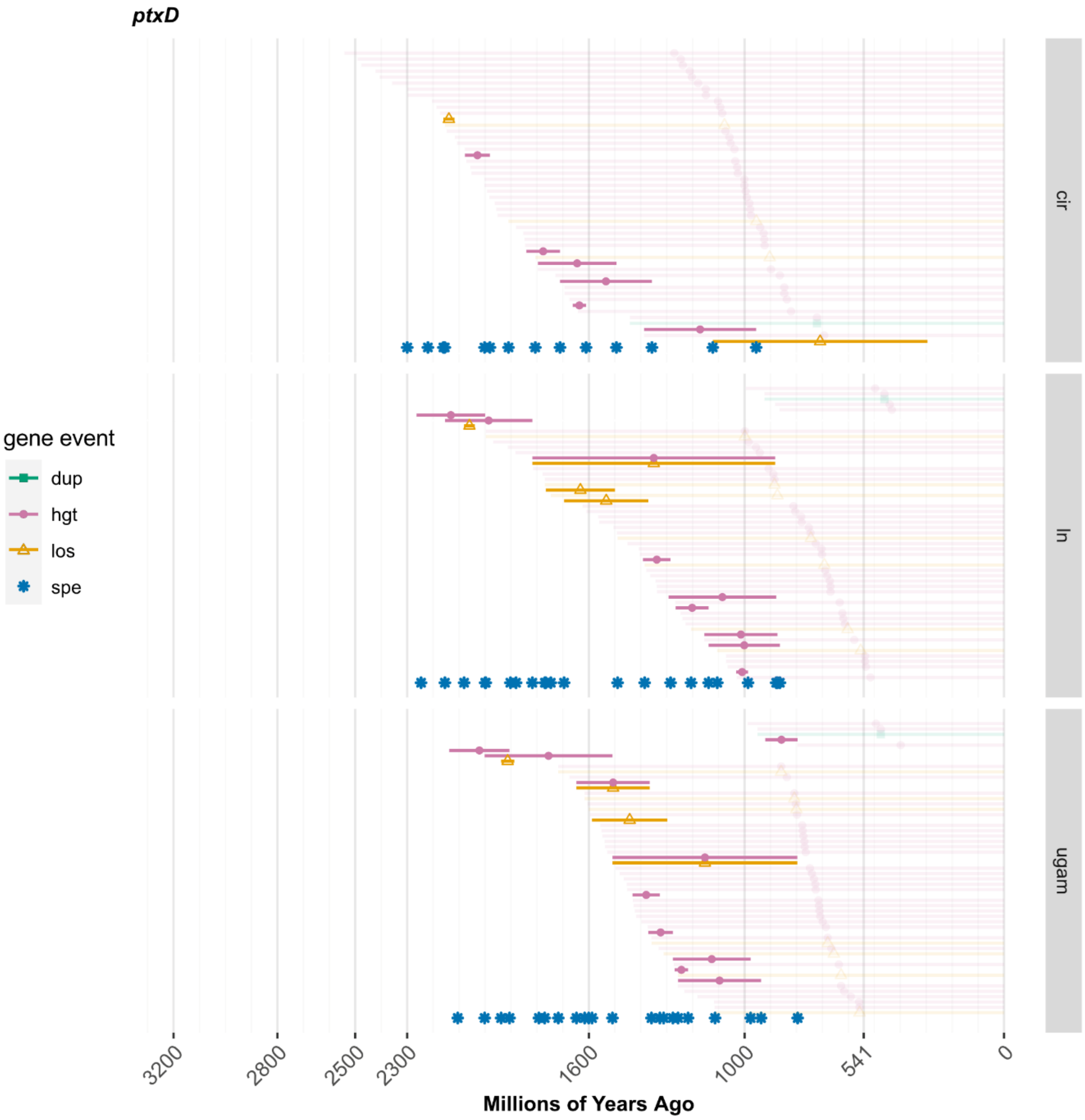

Figure 11: Uncertainty in estimating the origin of *ptxD*. Horizontal lines represent the lengths of branches where gene duplications (green), horizontal gene transfers (pink) and losses (orange) are predicted to have occurred. The midpoint of each branch is marked with shapes of the same colour, representing duplications (filled squares), horizontal gene transfers (filled circles) and losses (empty triangles). Darkness indicates whether the event occurred on an internal (dark colour) or terminal (faded) branch of the tree of life. Gene speciations (blue asterisks) are not associated with branch lengths because they occur on internal nodes of the tree. Results found using three different clock models are shown (cir: Cox-Ingersoll-Ross, ln: lognormal, ugam: uncorrelated gamma multipliers).

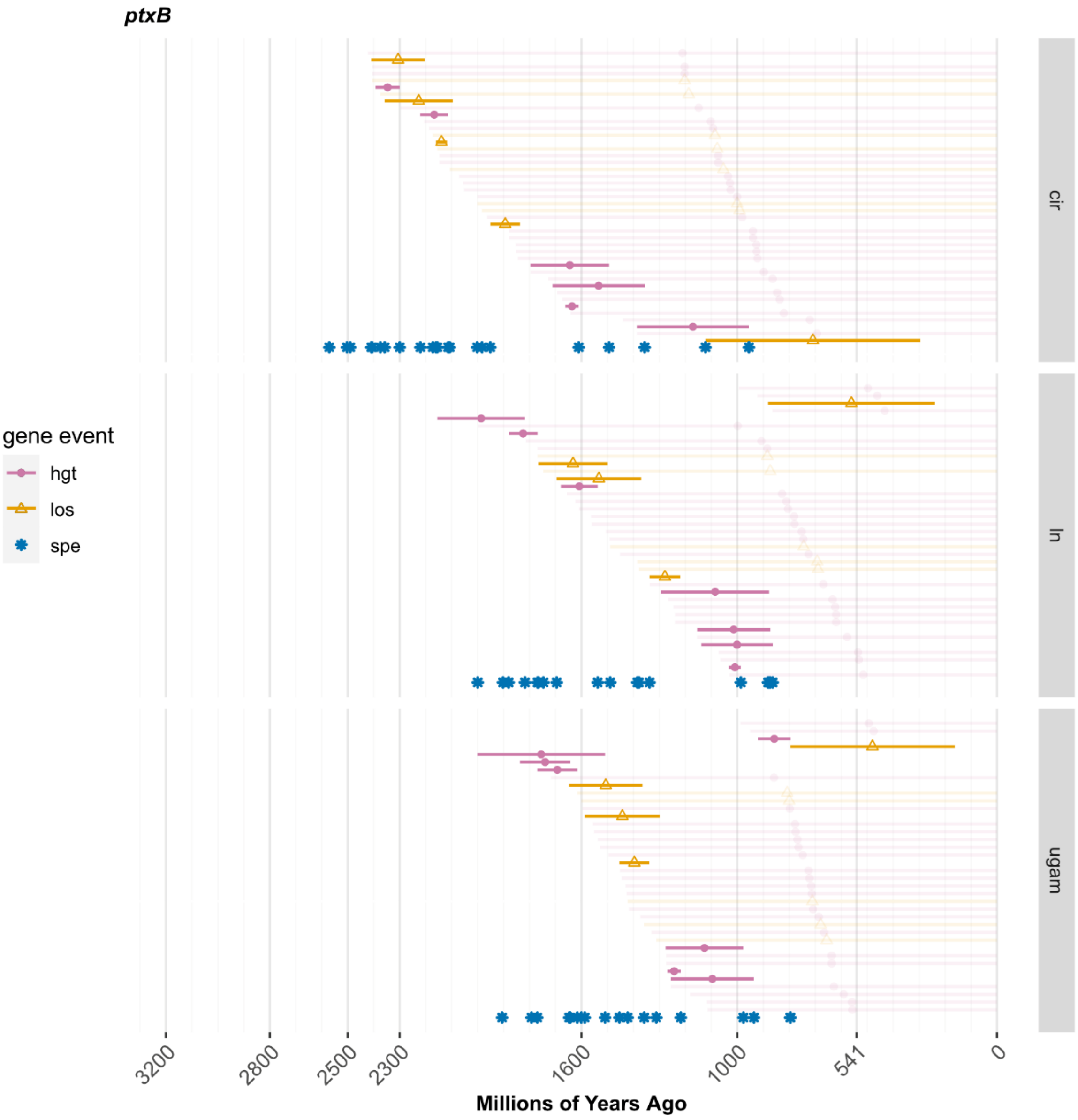

Figure 12: Uncertainty in estimating the origin of *ptxB*. Horizontal lines represent the lengths of branches where gene duplications (green), horizontal gene transfers (pink) and losses (orange) are predicted to have occurred. The midpoint of each branch is marked with shapes of the same colour, representing duplications (filled squares), horizontal gene transfers (filled circles) and losses (empty triangles). Darkness indicates whether the event occurred on an internal (dark colour) or terminal (faded) branch of the tree of life. Gene speciations (blue asterisks) are not associated with branch lengths because they occur on internal nodes of the tree. Results found using three different clock models are shown (cir: Cox-Ingersoll-Ross, ln: lognormal, ugam: uncorrelated gamma multipliers).

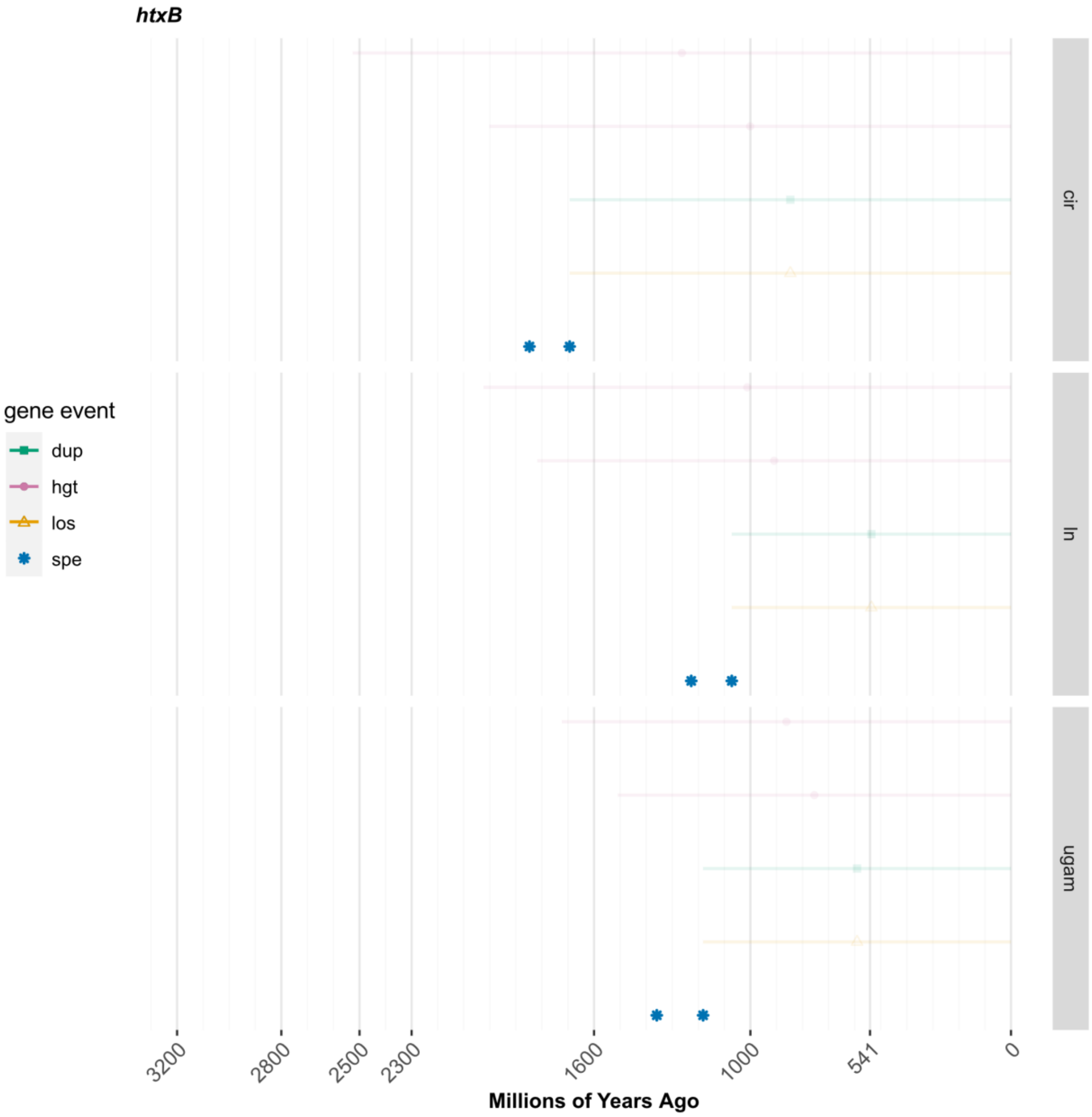

Figure 13: Uncertainty in estimating the origin of *htxB*. Horizontal lines represent the lengths of branches where gene duplications (green), horizontal gene transfers (pink) and losses (orange) are predicted to have occurred. The midpoint of each branch is marked with shapes of the same colour, representing duplications (filled squares), horizontal gene transfers (filled circles) and losses (empty triangles). Darkness indicates whether the event occurred on an internal (dark colour) or terminal (faded) branch of the tree of life. Gene speciations (blue asterisks) are not associated with branch lengths because they occur on internal nodes of the tree. Results found using three different clock models are shown (cir: Cox-Ingersoll-Ross, ln: lognormal, ugam: uncorrelated gamma multipliers).

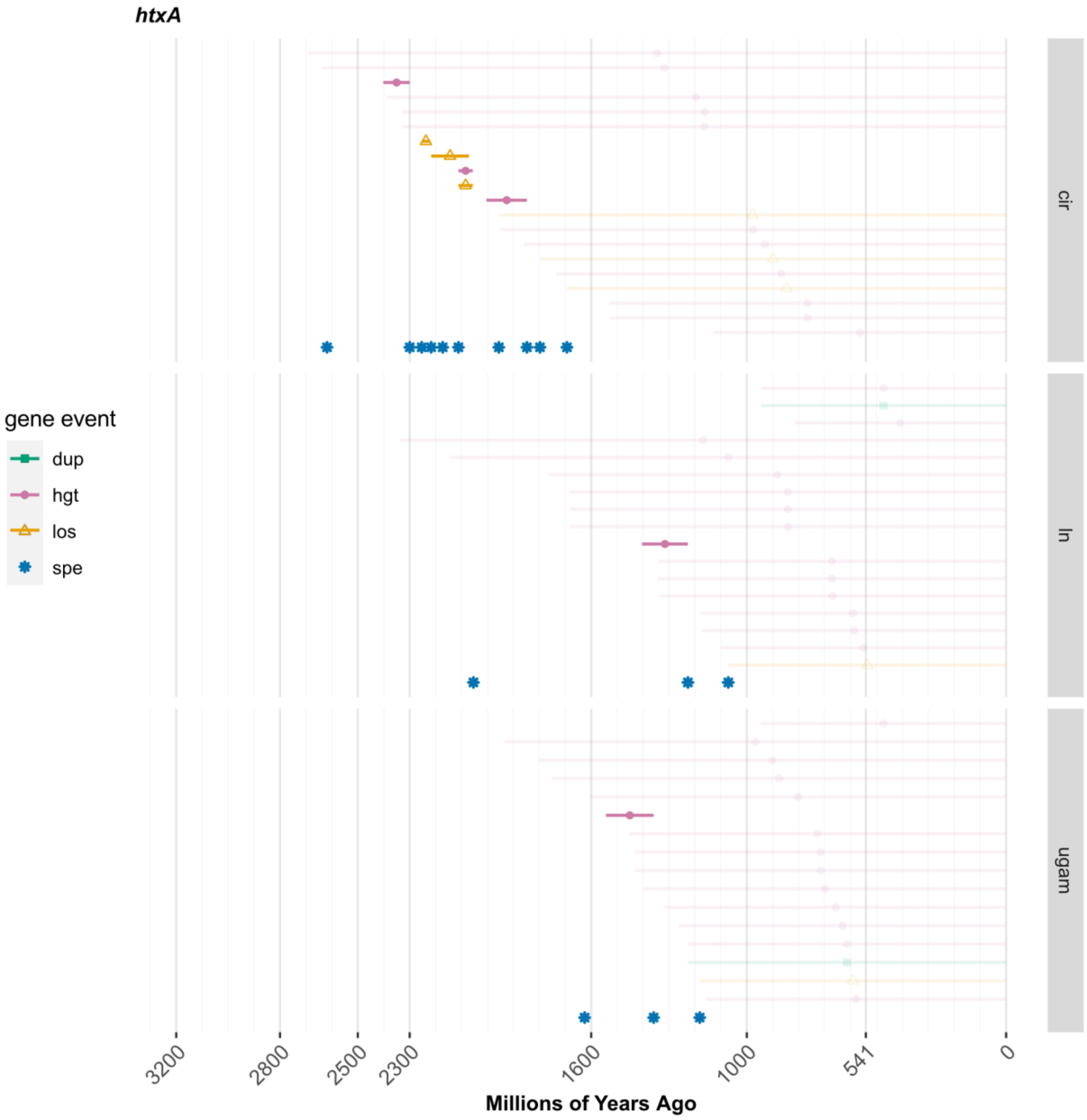

Figure 14: Uncertainty in estimating the origin of *htxA*. Horizontal lines represent the lengths of branches where gene duplications (green), horizontal gene transfers (pink) and losses (orange) are predicted to have occurred. The midpoint of each branch is marked with shapes of the same colour, representing duplications (filled squares), horizontal gene transfers (filled circles) and losses (empty triangles). Darkness indicates whether the event occurred on an internal (dark colour) or terminal (faded) branch of the tree of life. Gene speciations (blue asterisks) are not associated with branch lengths because they occur on internal nodes of the tree. Results found using three different clock models are shown (cir: Cox-Ingersoll-Ross, ln: lognormal, ugam: uncorrelated gamma multipliers).

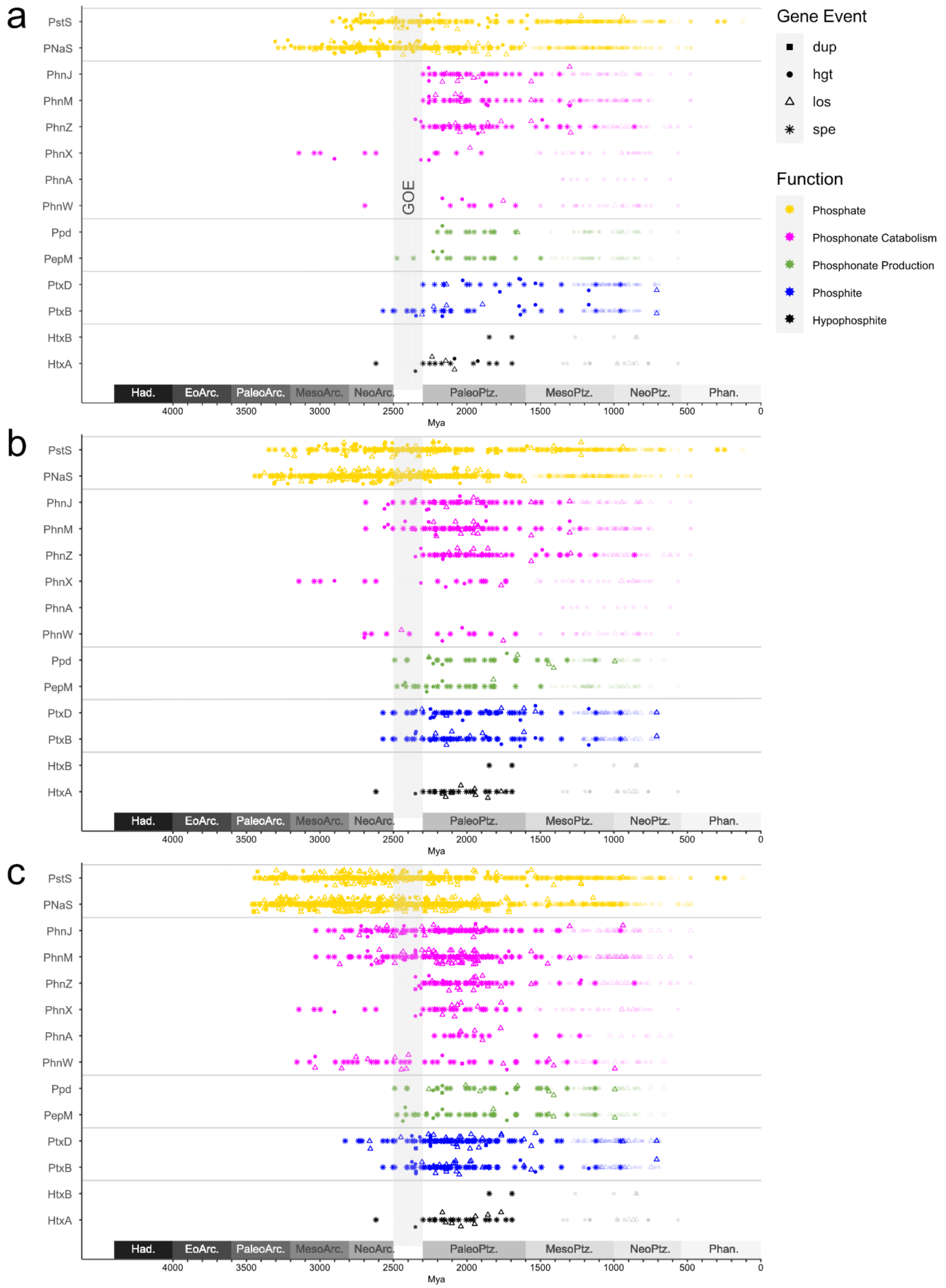

Figure 15: Effect of higher costs for horizontal gene transfer (HGT) on the estimated timing of evolution of phosphorus-cycling enzymes. HGT cost is varied from the default of 3 (a) to 4 (b) and 6 (c) whilst retaining the default costs of gene duplication, loss, and speciation. These reconciliations were calculated using results of molecular clocks made with the CIR clock model. Colours represent duplications (squares), transfers (circles),

losses (triangles) and speciations (asterisks) of genes associated with microbial phosphate import (yellow), phosphonate catabolism (pink), phosphonate production (green), phosphite import and oxidation (blue) and hypophosphite import and oxidation (black). Faded colours represent the midpoint of events which occur on terminal branches, whilst bright colours represent the midpoint of events which occur on internal branches. Mya, million years ago; GOE, great oxygenation event.

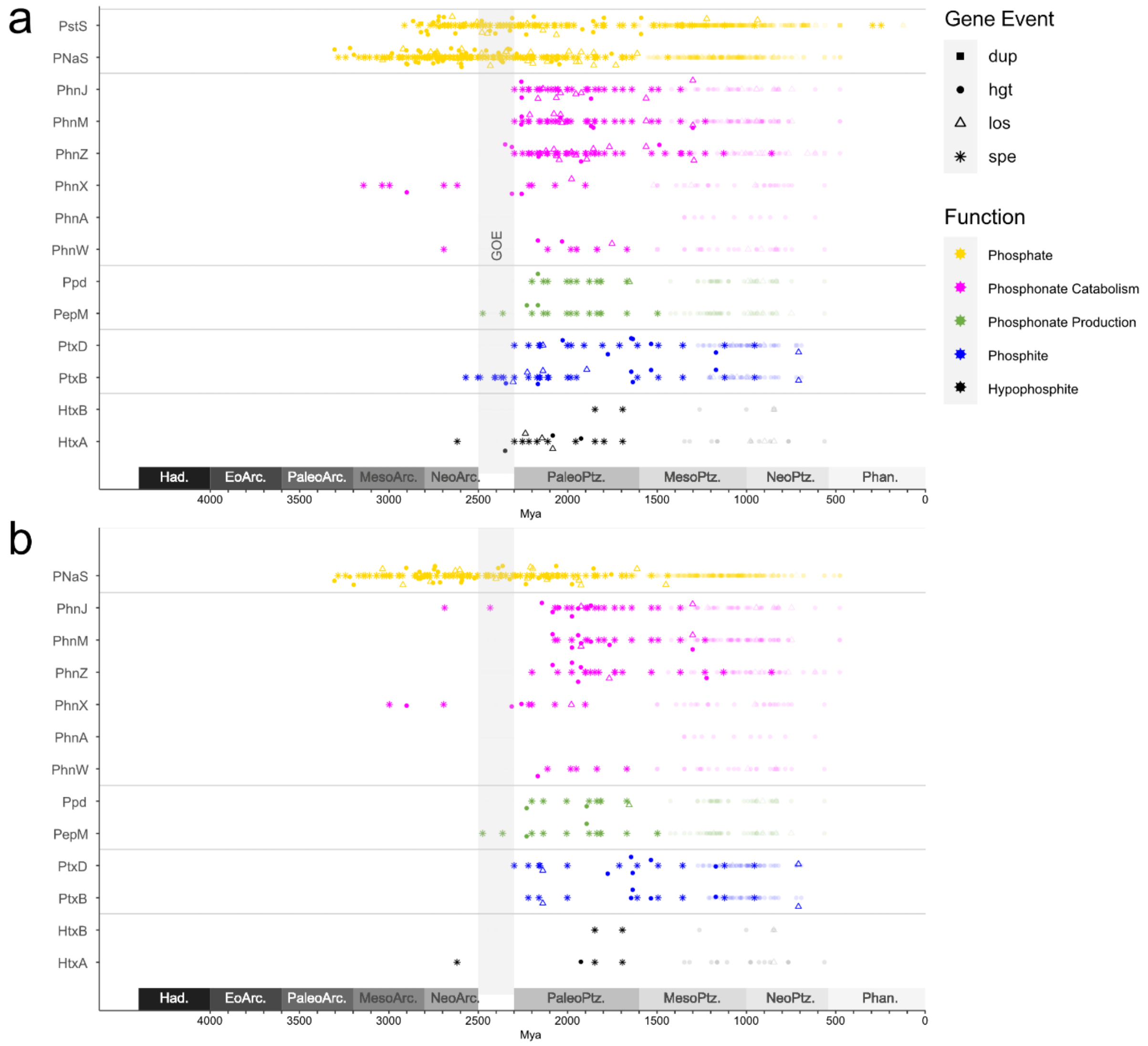

Figure 16: Effect of lower HGT costs on the estimated timing of the evolution of phosphorus-cycling enzymes. HGT cost is reduced from the default of 3 (a) down to 2 (b) whilst retaining the default costs of gene duplication, loss, and speciation. These reconciliations were calculated using results of molecular clocks made with the CIR clock model. Colours represent duplications (squares), transfers (circles), losses (triangles) and speciations (asterisks) of genes associated with microbial phosphate import (yellow), phosphonate catabolism (pink), phosphonate production (green), phosphite import and oxidation (blue) and hypophosphite import and oxidation (black). Faded colours represent the midpoint of events which occur on terminal branches, whilst bright colours represent the midpoint of events which occur on internal branches. Mya, million years ago; GOE, great oxygenation event.

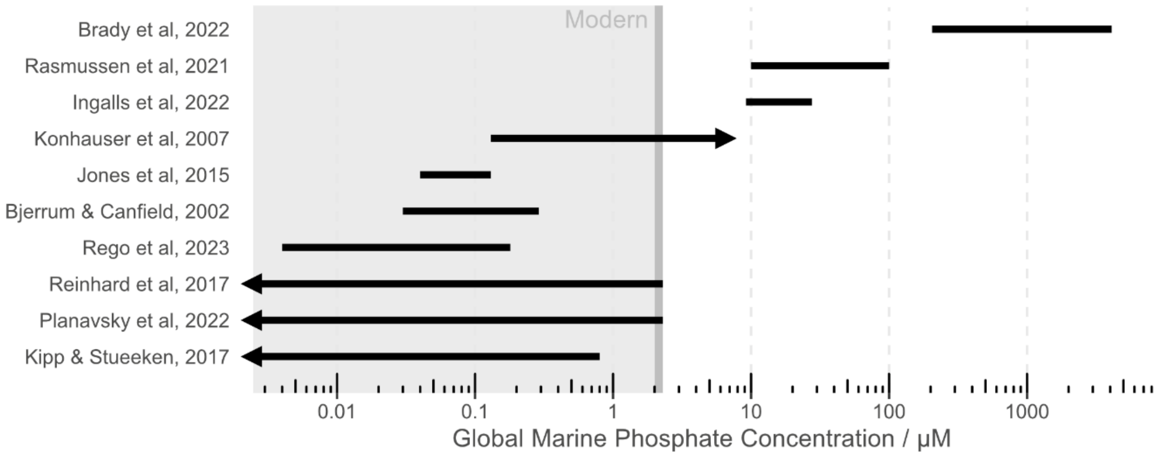

Figure 17: Geochemical estimates of phosphate concentrations in the Archaean ocean. Note how phosphate concentrations are indicated on a logarithmic x axis, made with base 10.

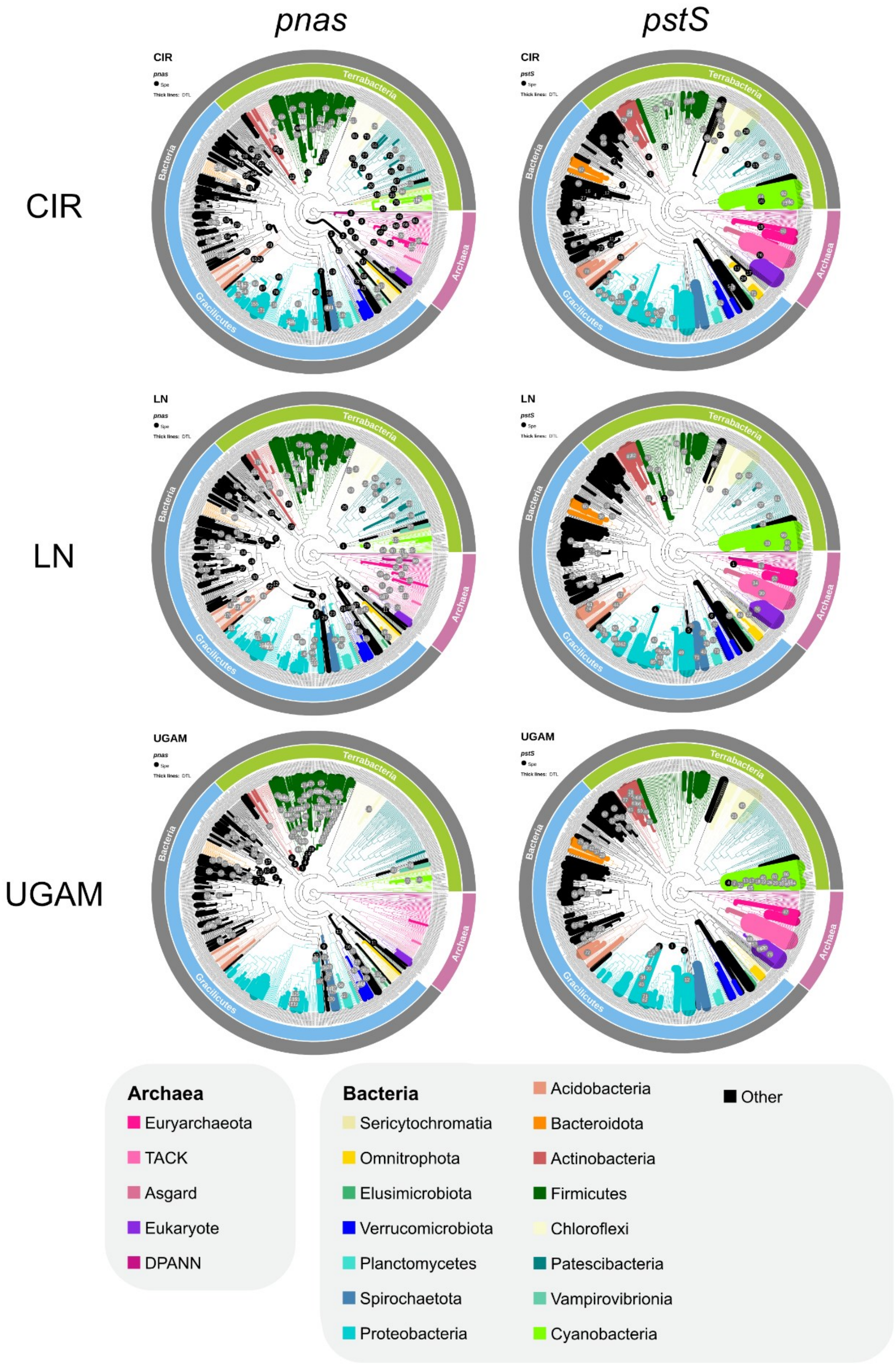

Figure 18: Phylogenetic trees representing where speciations (numbered circles), duplications, transfers and losses (thick lineages) of *pstS* (right) and *pnas* (left) occurred in the tree of life based on three different molecular clock models. Speciations are numbered in chronological order which Archaean events highlighted in black circles, and others in grey circles. The thickness of each branch corresponds to the number of duplications, transfers and losses that occurred on the branch, whereas branch colours represent which phyla the lineage belongs to.

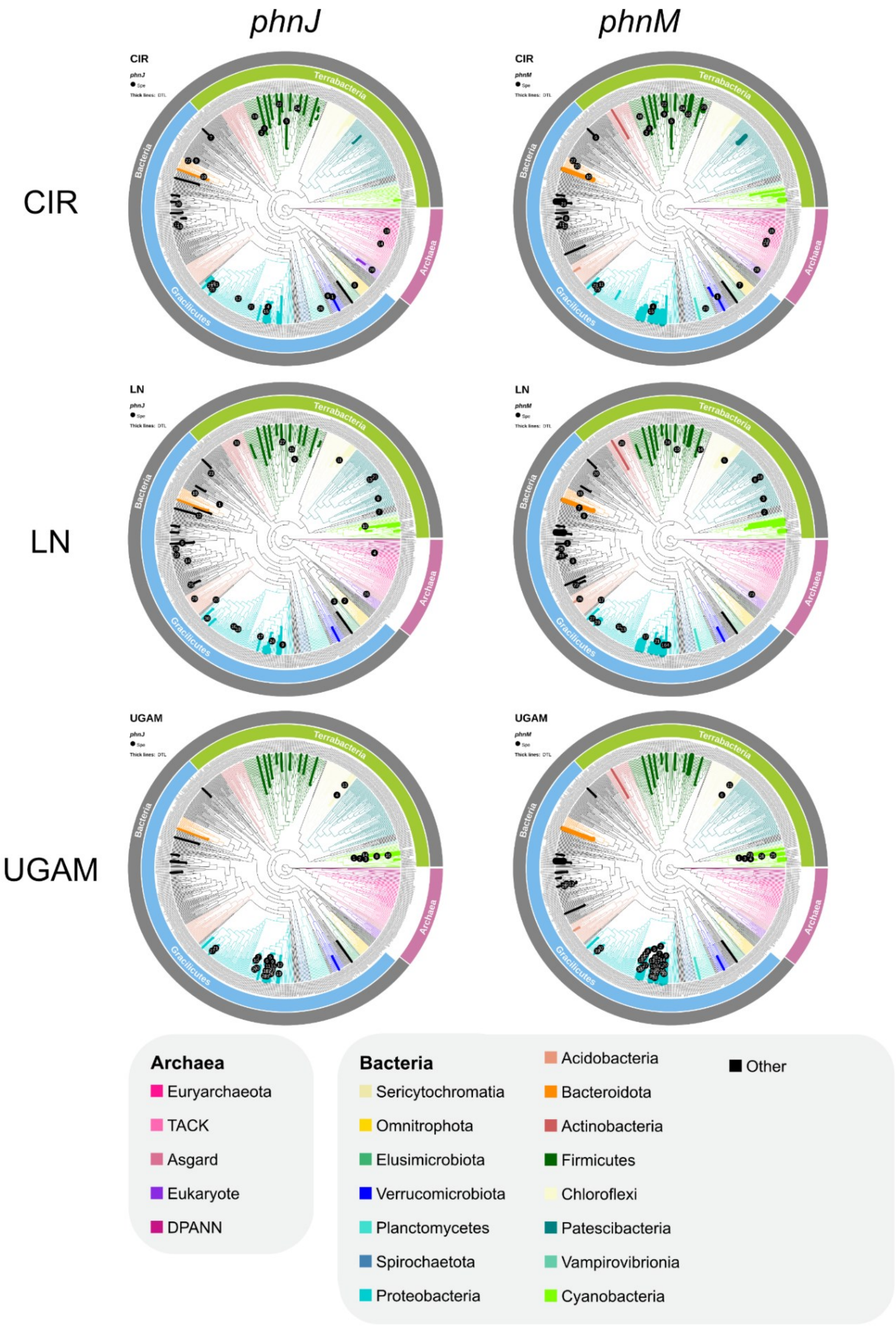

Figure 19: Phylogenetic trees representing where speciations (black numbered circles), duplications, transfers and losses (thick lineages) of *phnJ* (left) and *phnM* (right) occurred in the tree of life based on three different molecular clock models. Speciations are numbered in chronological order. The thickness of each branch corresponds to the number of duplications, transfers and losses that occurred on the branch, whereas branch colours represent which phyla the lineage belongs to.

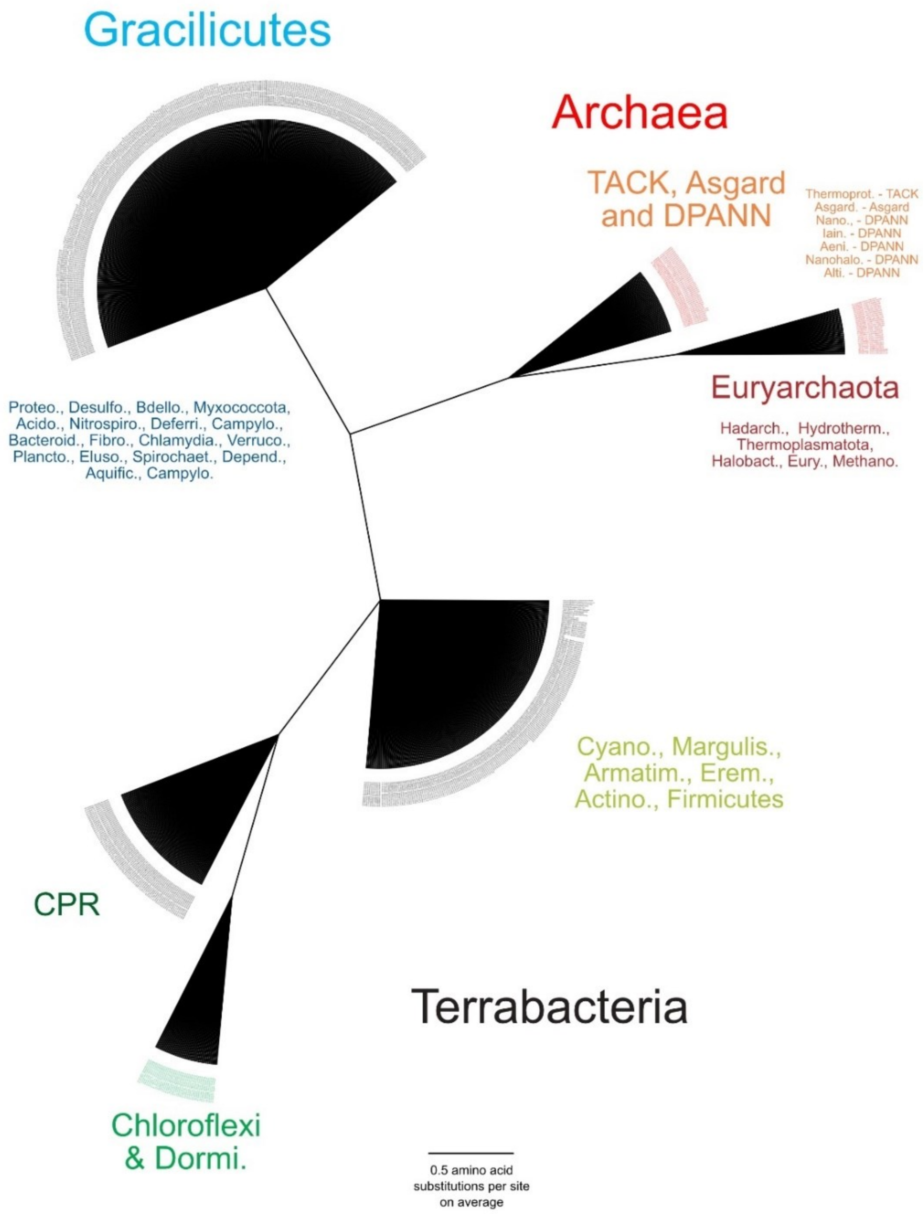

Figure 20: Constraints applied to the tree of life and molecular clock. Coloured text next to wedges describes which phyla they contain, including Archaea (red and orange), bacteria of the Gracilicutes clade (blue) and bacteria of the Terrabacteria clade (green). The constraint ensures that Euryarchaeota are more closely-related to each other than they are to other Archaea to match the findings of previous Archaeal phylogenies [4,5]. Similarly, Gracilicutes are constrained to be more closely related to each other than to Terrabacteria or Archaea and vice versa based on the findings of [6-13]. Although previous research (e.g. [8-10,14,15]) produces conflicting evidence on the monophyly of Terrabacteria, we chose to constrain them here because tree certainty metrics have found that artefacts in the form of long-branch attraction towards Archaea and uneven taxon sampling can split the Terrabacterial phylum into separate parts [11]. CPR are assumed to be closely-related to Chloroflexi and Dormibacteriota within the Terrabacteria despite some conflicting evidence (e.g. [9,10,14]) because their placement outside of Terrabacteria has also been found to be a result of uneven taxon sampling [6-8,11].

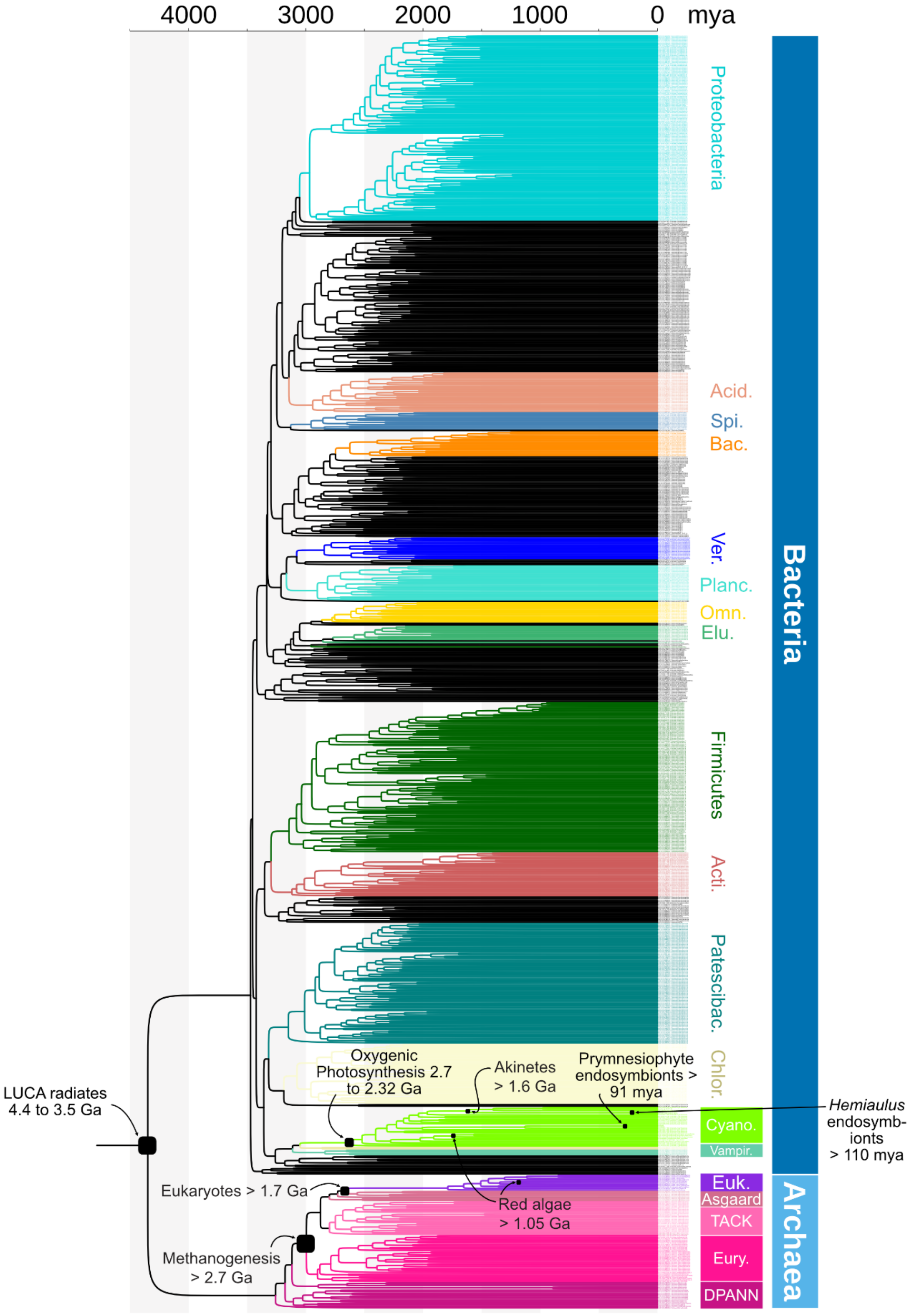

Figure 21: Calibrations for molecular clock analyses. The position of each calibration point are annotated in black text over a time-calibrated tree made with the CIR clock model. Coloured branches indicate different bacterial and archaeal phyla labelled in text of the same colour. Ultrafast bootstrap support values are presented in the species tree on the open science framework repository, https://osf.io/vt5rw/?view_only=b13a53f4d87c44d1a82a18b176523c5b. Acid. Acidobacteriota, Spi. Spirochaetota, Bac., Bacteroidota, Ver., Verrucomicrobiota, Planc. Planctomycetota, Omn. Omnitrophota, Elu. Elusimicrobiota, Acti. Actinobacteriota, Patescibac. Patescibacteria, Chlor. Chloroflexota, Cyano. Cyanobacteria, Vampiro. Vampirovibrionia, Euk. Eukaryota, Eury. Euryarchaeota.

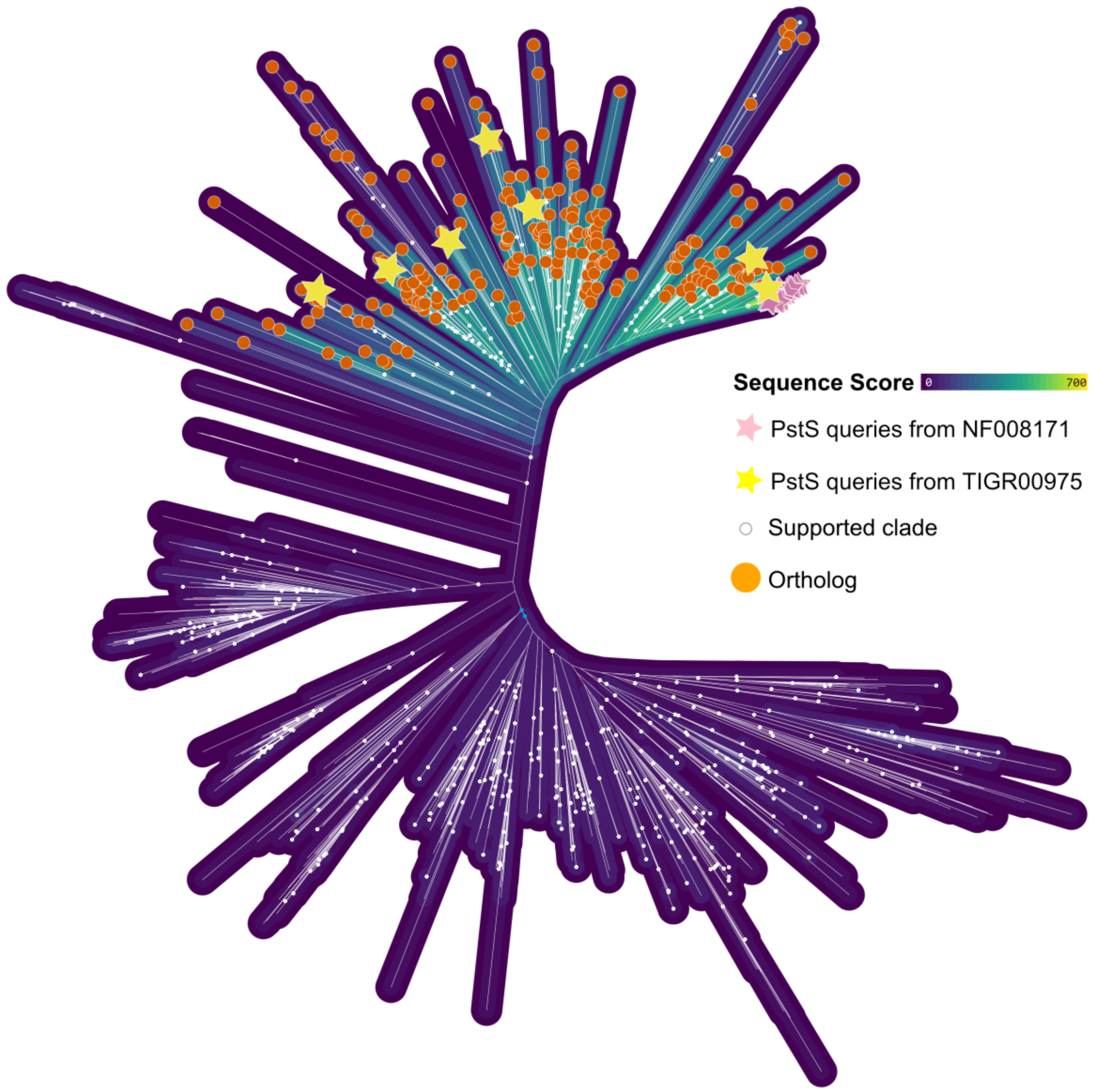

Figure 22: Identification of *pstS* orthologs. Maximum-likelihood phylogeny of *pstS* homologs, with bitscores represented by coloured outlines surrounding each branch. Orthologs (orange circles) were identified and selected for further analyses based on their bitscore and relationship to query sequences (yellow stars and pink stars) from the respective HMM profiles. White circles indicate ultrafast bootstrap support values => 95.

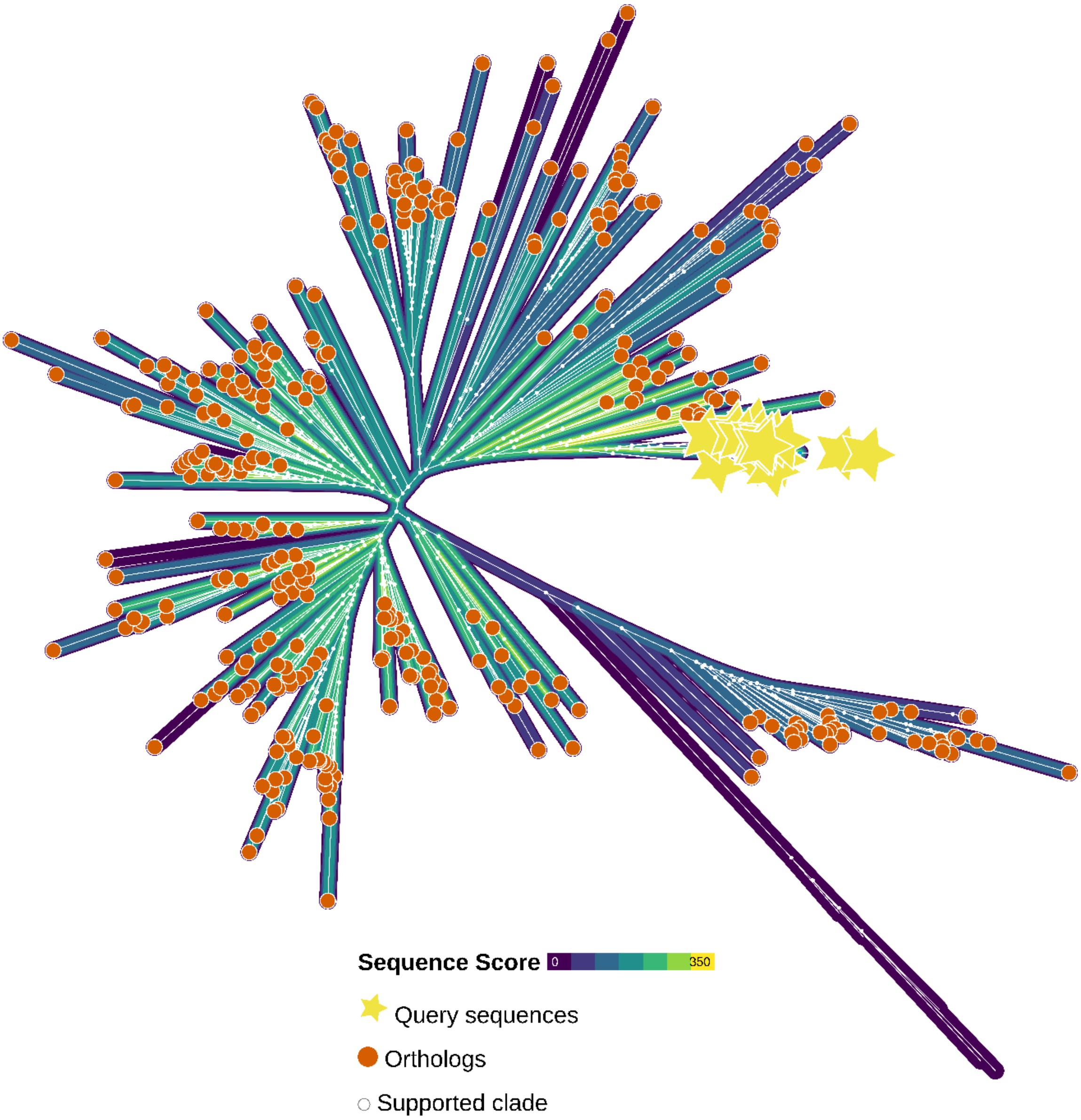

Figure 23: Identification of *pnas* orthologs. Maximum-likelihood phylogeny of *pnas* homologs, with bitscores represented by coloured outlines surrounding each branch. Orthologs (orange circles) were identified and selected for further analyses based on their bitscore and relationship to query sequences (yellow stars) from the respective HMM profiles. White circles indicate ultrafast bootstrap support values => 95.

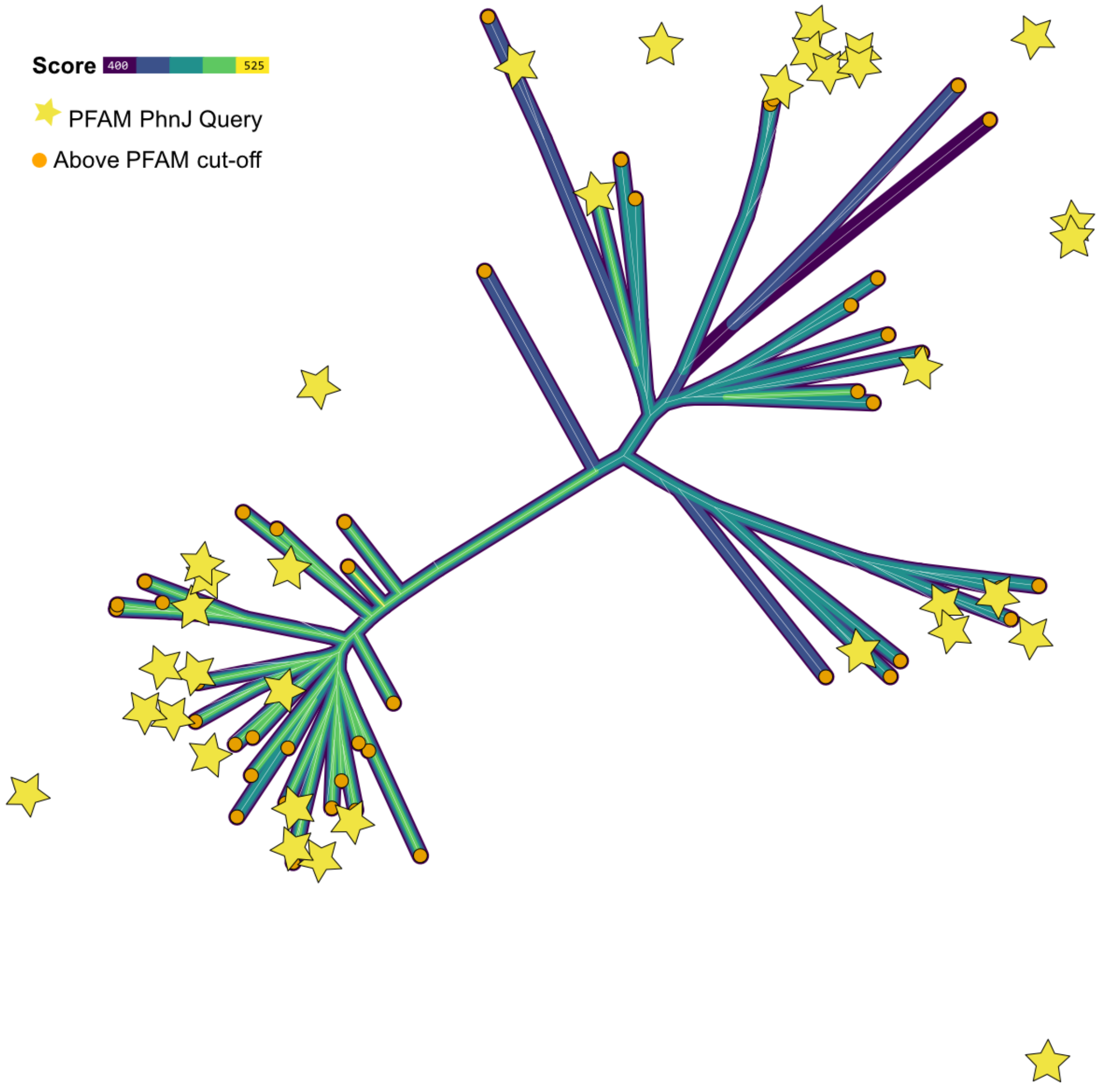

Figure 24: Identification of *phnJ* orthologs. Maximum-likelihood phylogeny of *phnJ* homologs, with bitscores represented by coloured outlines surrounding each branch. Orthologs (orange circles) were identified and selected for further analyses based on their bitscore and relationship to query sequences (yellow stars) from the respective HMM profiles. White circles indicate ultrafast bootstrap support values => 95.

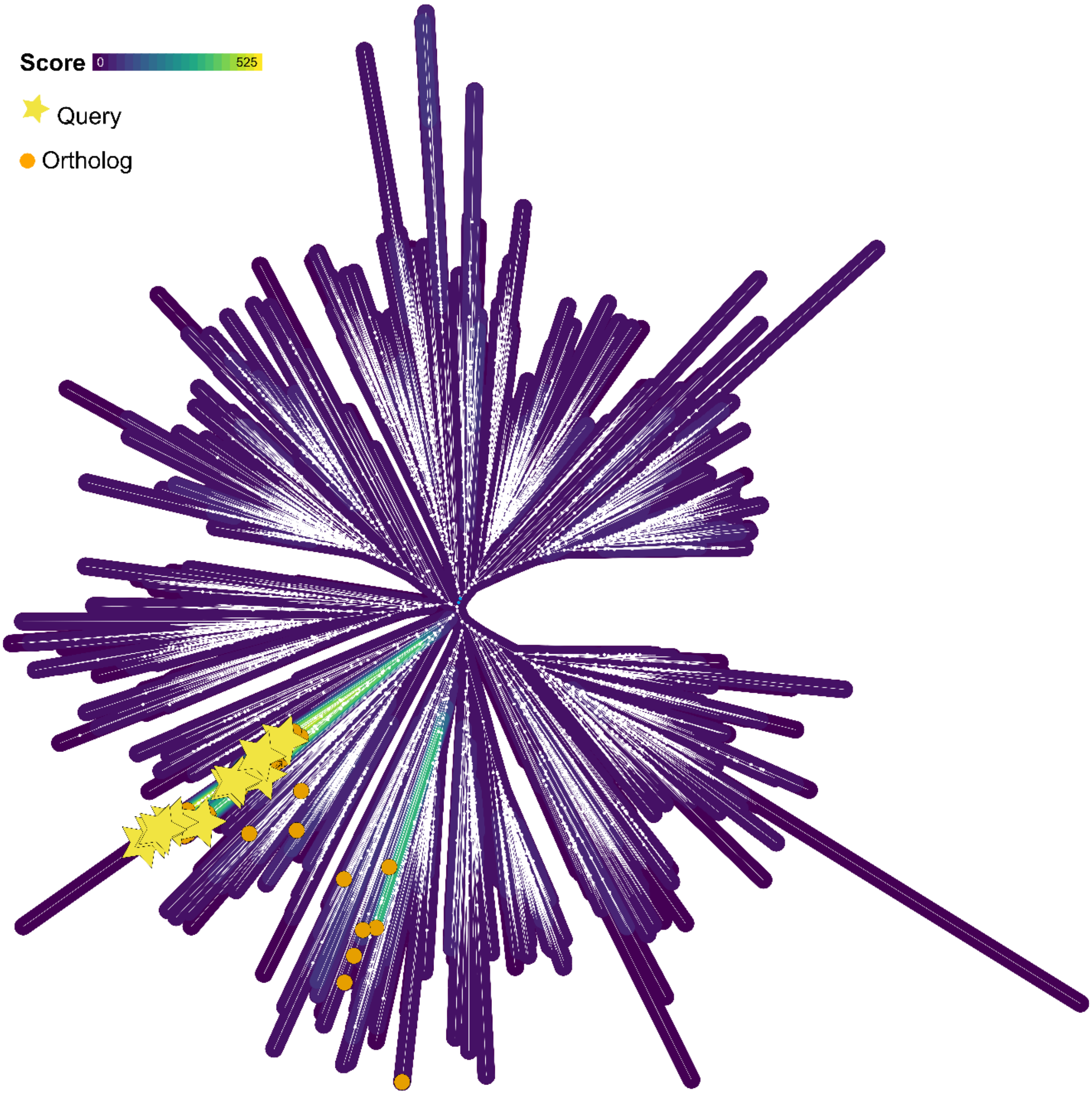

Figure 25: Identification of *phnM* orthologs. Maximum-likelihood phylogeny of *phnM* homologs, with bitscores represented by coloured outlines surrounding each branch. Orthologs (orange circles) were identified and selected for further analyses based on their bitscore and relationship to query sequences (yellow stars) from the respective HMM profiles. White circles indicate ultrafast bootstrap support values => 95.

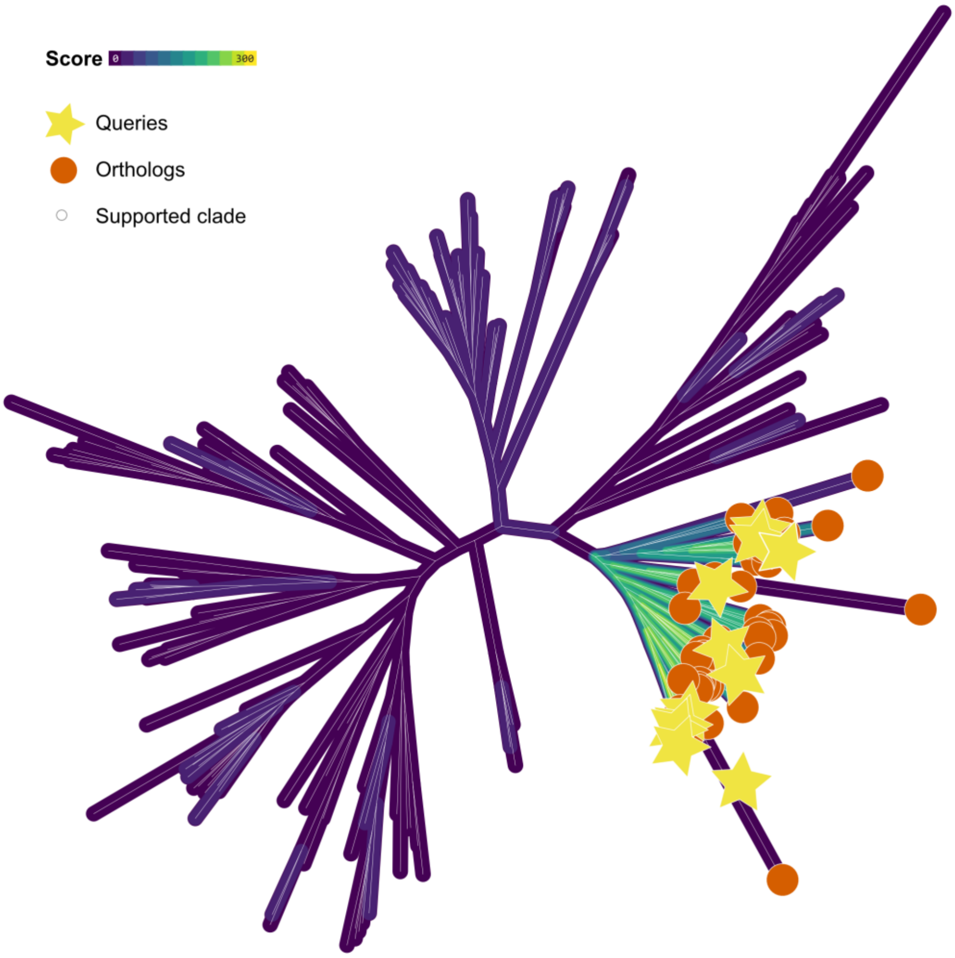

Figure 26: Identification of *phnZ* orthologs. Maximum-likelihood phylogeny of *phnZ* homologs, with bitscores represented by coloured outlines surrounding each branch. Orthologs (orange circles) were identified and selected for further analyses based on their bitscore and relationship to query sequences (yellow stars) from the respective HMM profiles. White circles indicate ultrafast bootstrap support values => 95.

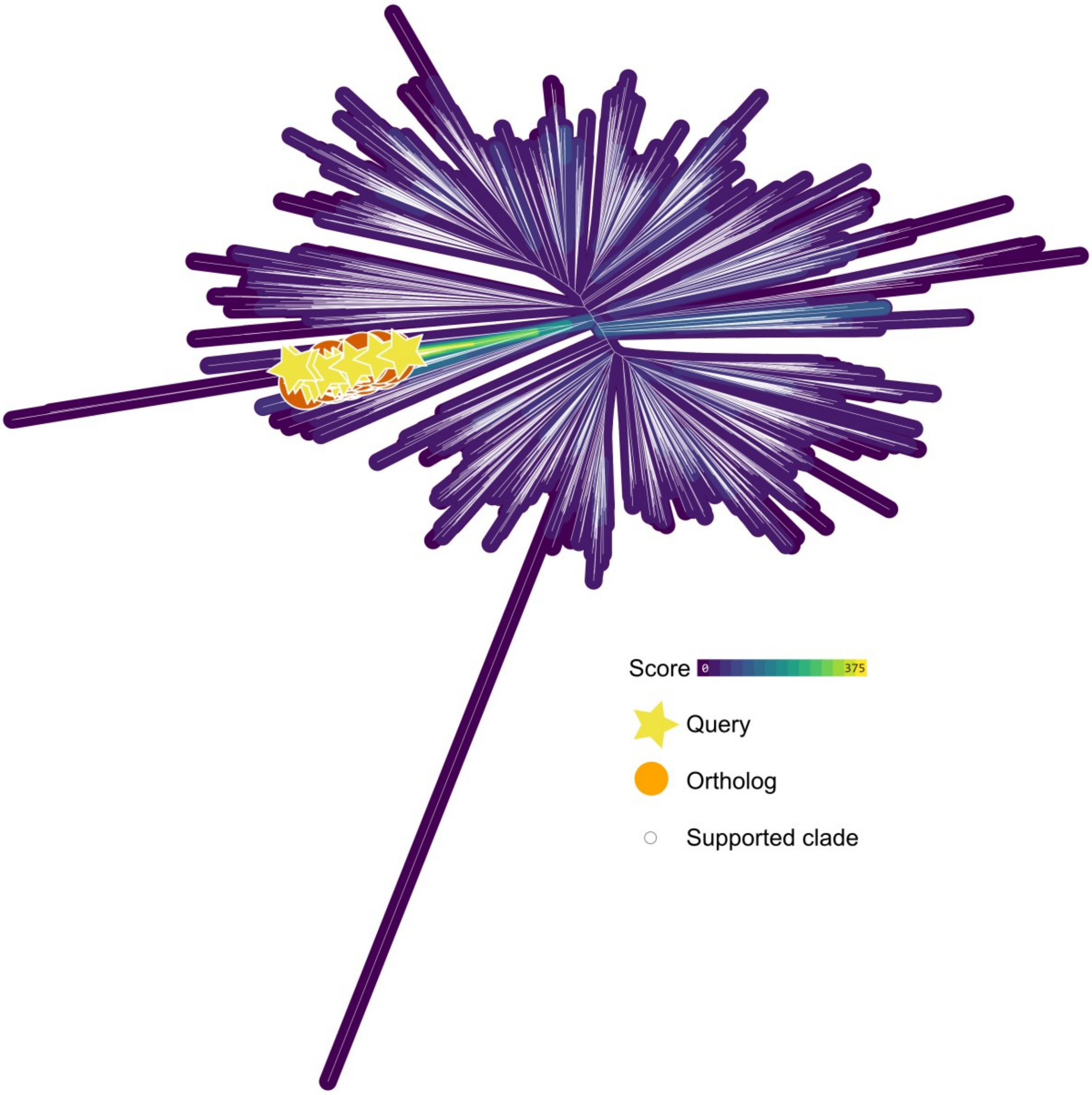

Figure 27: Identification of *phnX* orthologs. Maximum-likelihood phylogeny of *phnX* homologs, with bitscores represented by coloured outlines surrounding each branch. Orthologs (orange circles) were identified and selected for further analyses based on their bitscore and relationship to query sequences (yellow stars) from the respective HMM profiles. White circles indicate ultrafast bootstrap support values => 95.

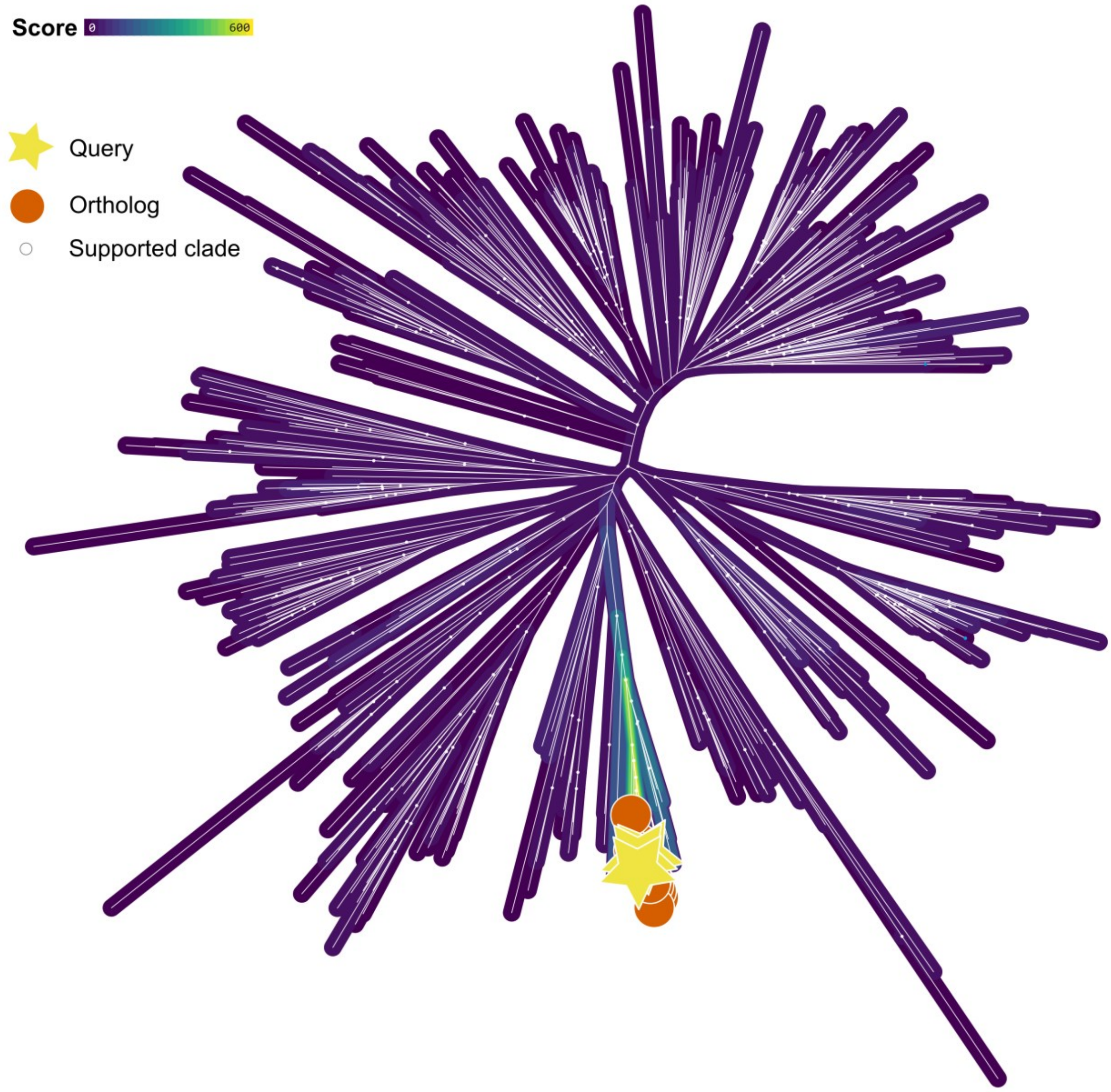

Figure 28: Identification of *phnA* orthologs. Maximum-likelihood phylogeny of *phnA* homologs, with bitscores represented by coloured outlines surrounding each branch. Orthologs (orange circles) were identified and selected for further analyses based on their bitscore and relationship to query sequences (yellow stars) from the respective HMM profiles. White circles indicate ultrafast bootstrap support values => 95.

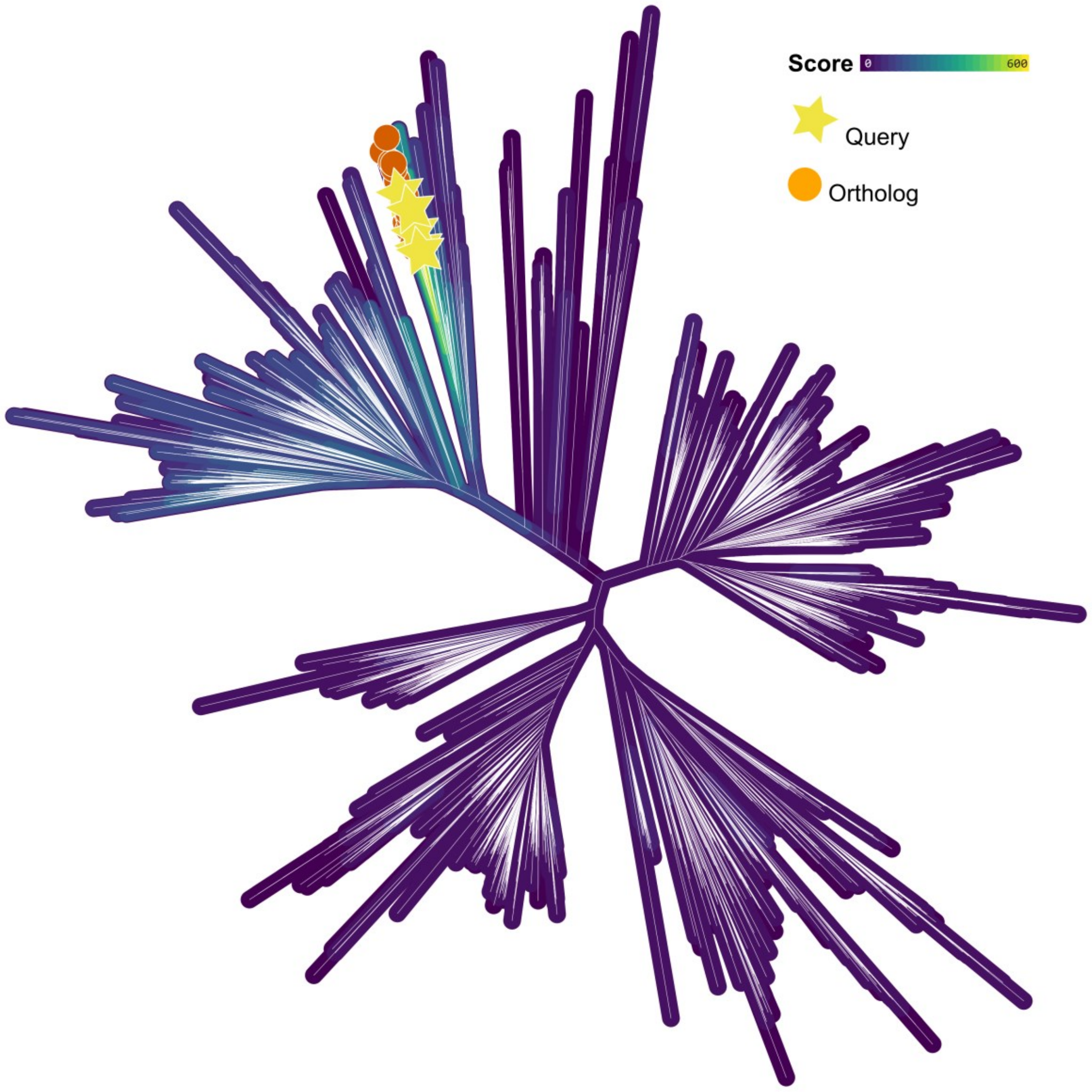

Figure 29: Identification of *phnW* orthologs. Maximum-likelihood phylogeny of *phnW* homologs, with bitscores represented by coloured outlines surrounding each branch. Orthologs (orange circles) were identified and selected for further analyses based on their bitscore and relationship to query sequences (yellow stars) from the respective HMM profiles.

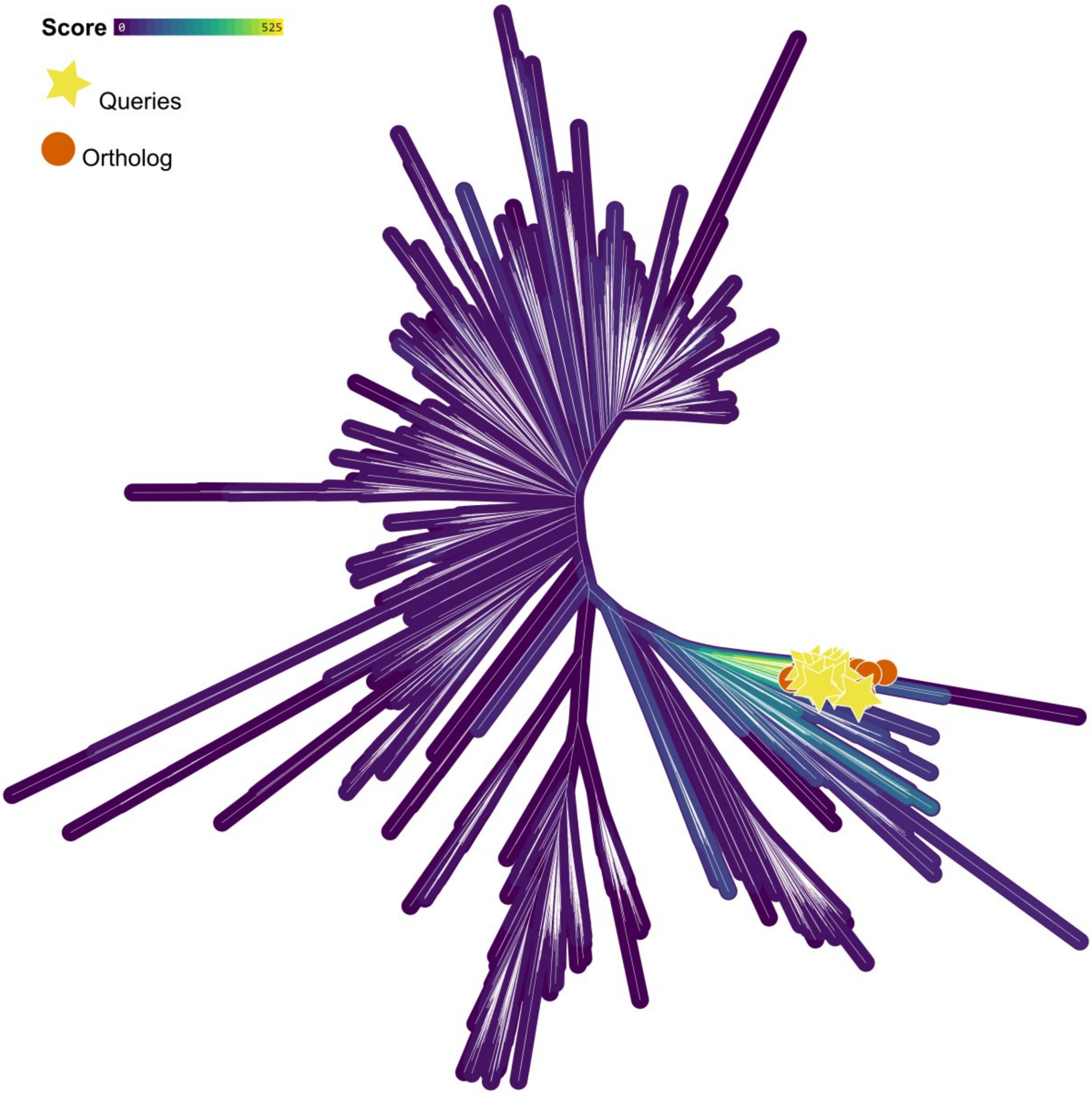

Figure 30: Identification of *ppd* orthologs. Maximum-likelihood phylogeny of *ppd* homologs, with bitscores represented by coloured outlines surrounding each branch. Orthologs (orange circles) were identified and selected for further analyses based on their bitscore and relationship to query sequences (yellow stars) from the respective HMM profiles.

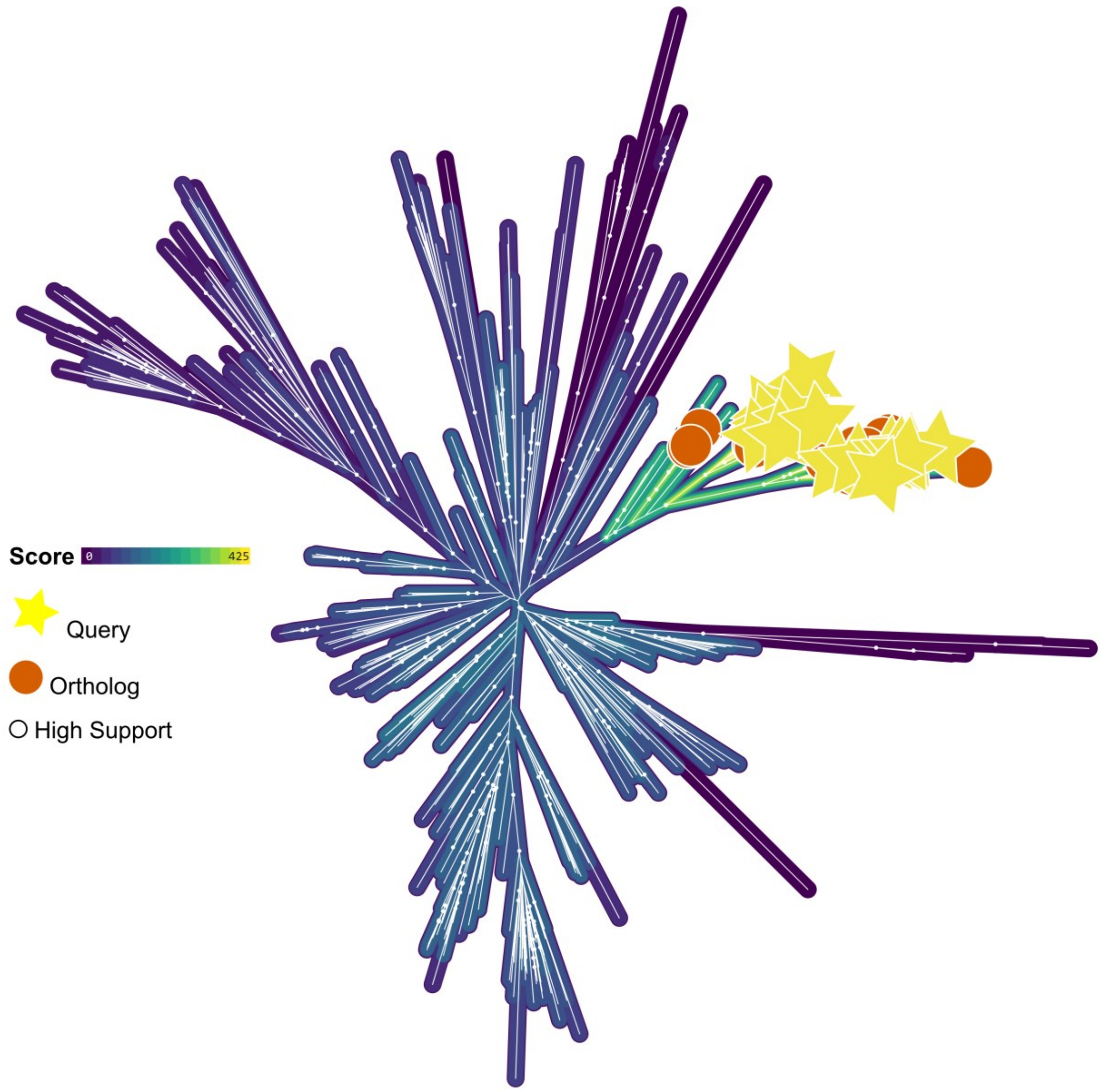

Figure 31: Identification of *pepM* orthologs. Maximum-likelihood phylogeny of *pepM* homologs, with bitscores represented by coloured outlines surrounding each branch. Orthologs (orange circles) were identified and selected for further analyses based on their bitscore and relationship to query sequences (yellow stars) from the respective HMM profiles. White circles indicate ultrafast bootstrap support values => 95.

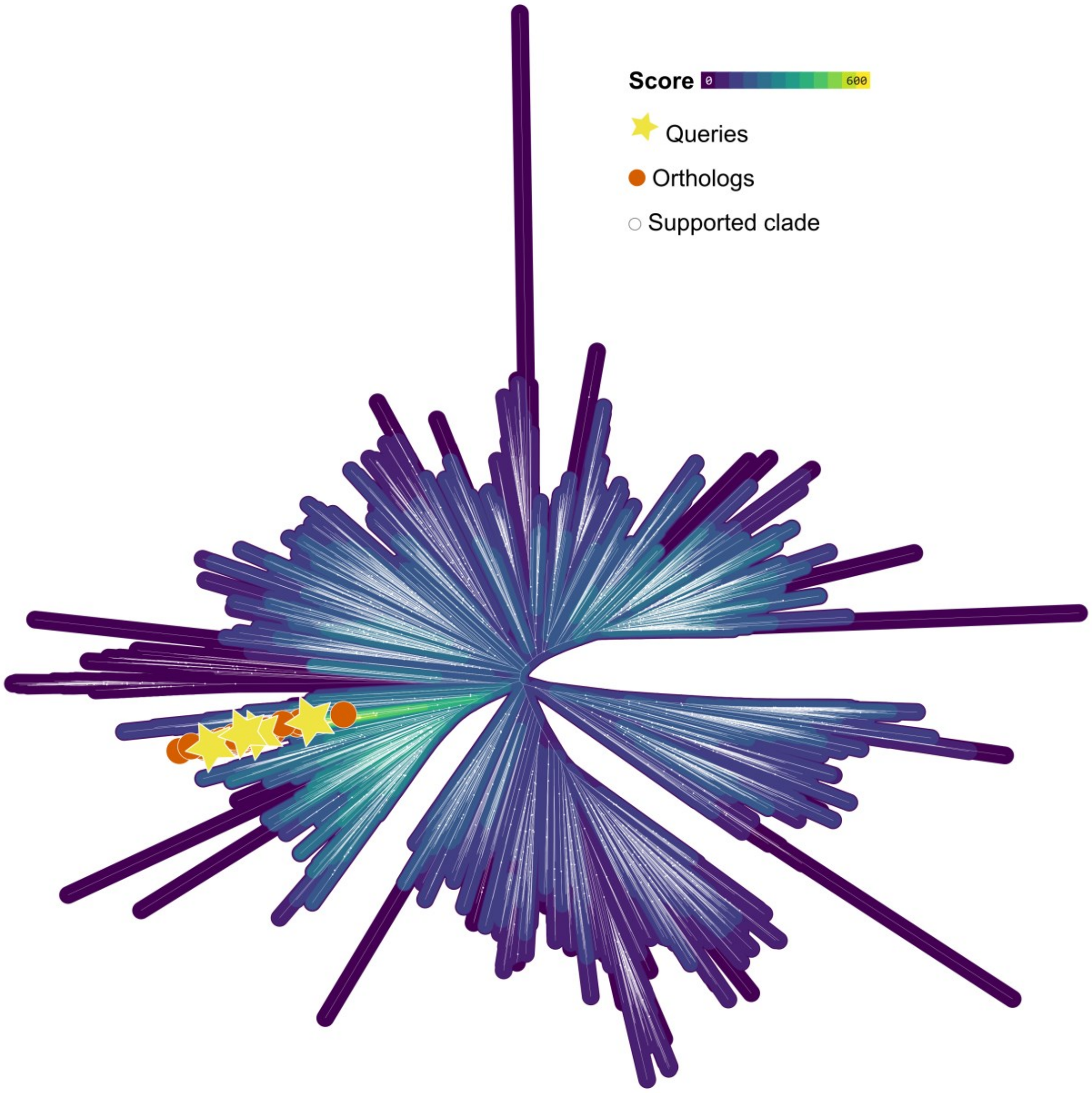

Figure 32: Identification of *ptxD* orthologs. Maximum-likelihood phylogeny of *ptxD* homologs, with bitscores represented by coloured outlines surrounding each branch. Orthologs (orange circles) were identified and selected for further analyses based on their bitscore and relationship to query sequences (yellow stars) from the respective HMM profiles. White circles indicate ultrafast bootstrap support values => 95.

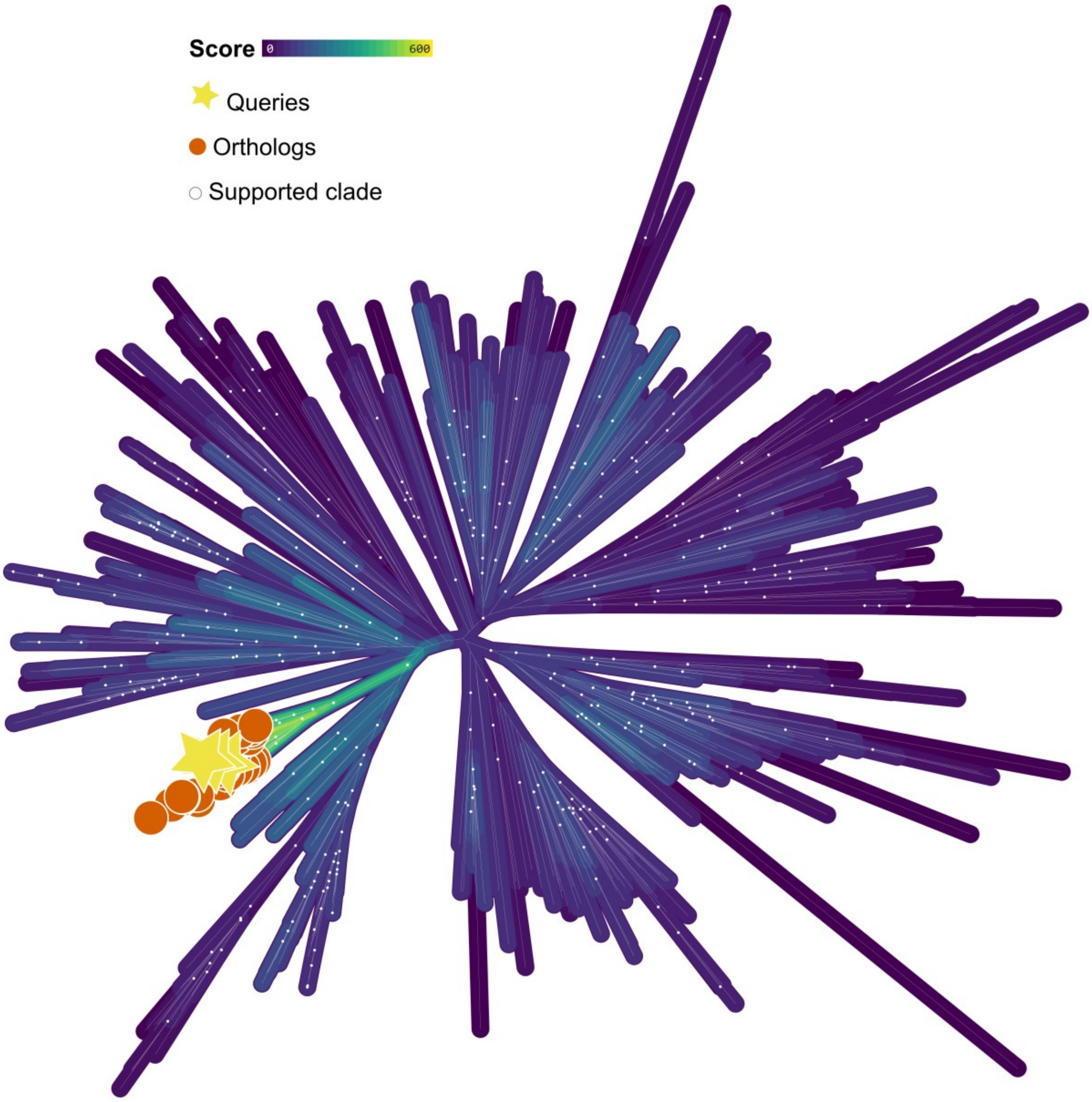

Figure 33: Identification of *ptxB* orthologs. Maximum-likelihood phylogeny of *ptxB* homologs, with bitscores represented by coloured outlines surrounding each branch. Orthologs (orange circles) were identified and selected for further analyses based on their bitscore and relationship to query sequences (yellow stars) from the respective HMM profiles. White circles indicate ultrafast bootstrap support values => 95.

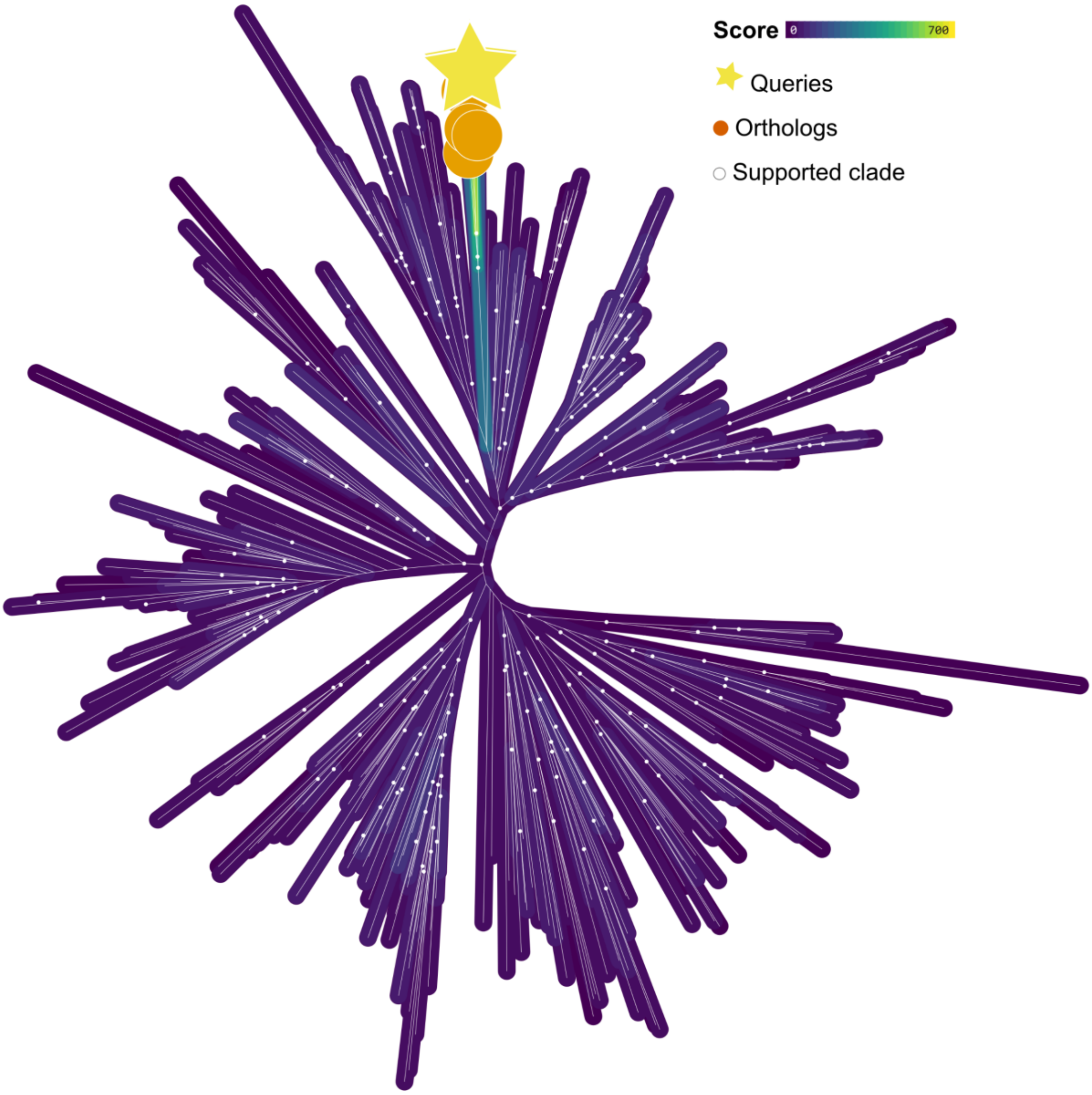

Figure 34: Identification of *htxB* orthologs. Maximum-likelihood phylogeny of *htxB* homologs, with bitscores represented by coloured outlines surrounding each branch. Orthologs (orange circles) were identified and selected for further analyses based on their bitscore and relationship to query sequences (yellow stars) from the respective HMM profiles. White circles indicate ultrafast bootstrap support values => 95.

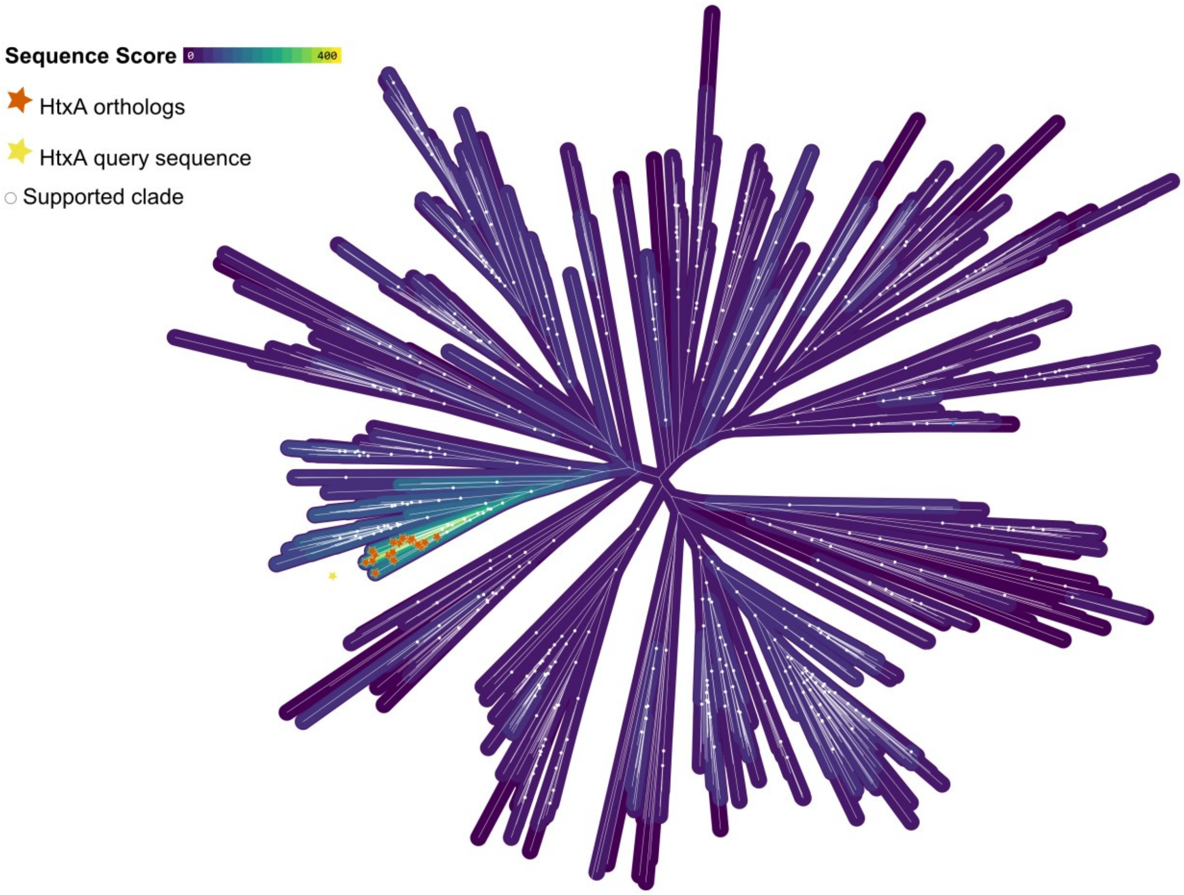

Figure 35: Identification of *htxA* orthologs. Maximum-likelihood phylogeny of *htxA* homologs, with bitscores represented by coloured outlines surrounding each branch. Orthologs (orange stars) were identified and selected for further analyses based on their bitscore and relationship to query sequences (yellow stars) from the respective HMM profiles. White circles indicate ultrafast bootstrap support values => 95.

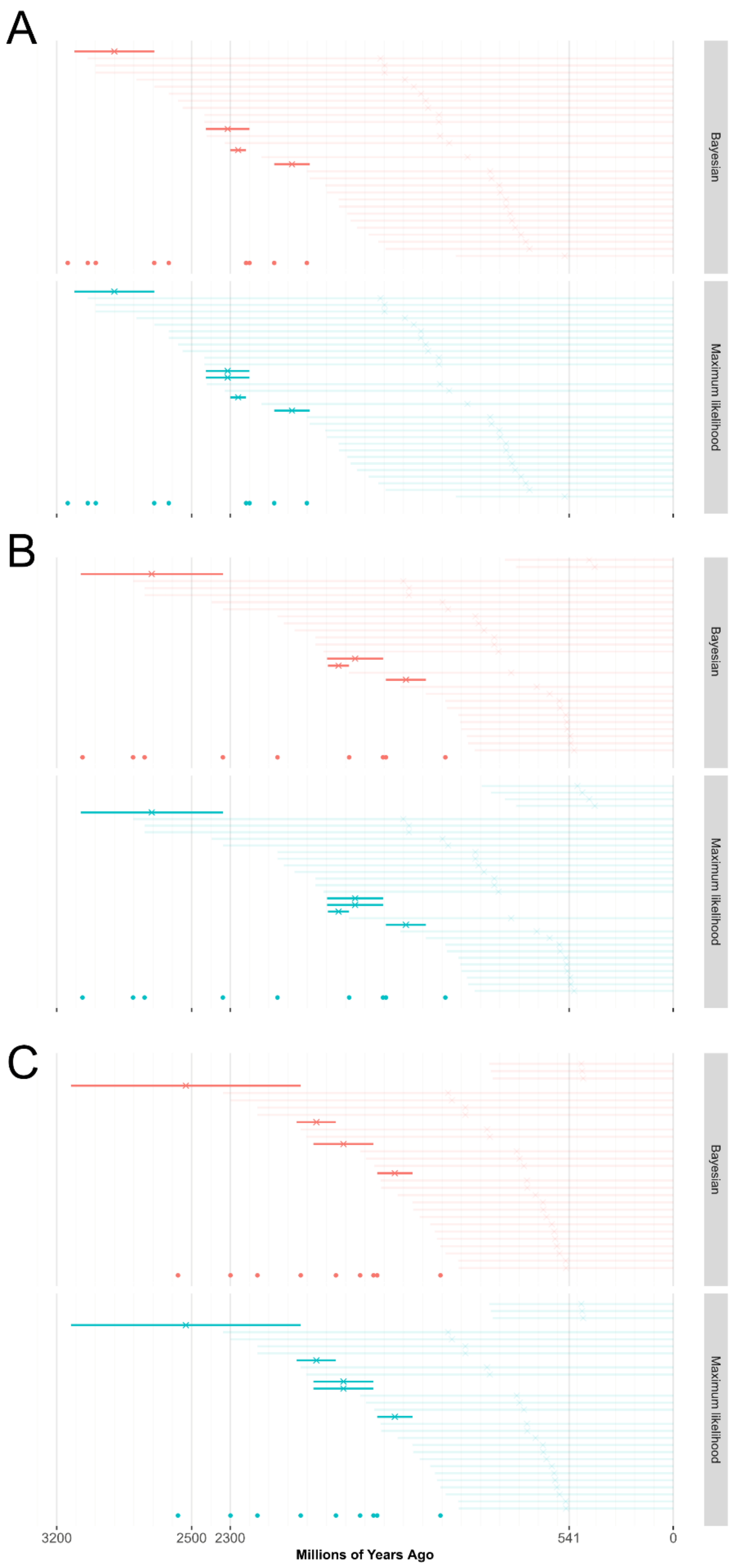

Figure 36: Differences between estimating the origin of *phnX* using cir (A), ln (B) and ugam (c) with Bayesian and Maximum Likelihood methodology. Bayesian results (orange) were collected using MrBayes to reconstruct the *phnX* phylogeny, whereas maximum likelihood results (cyan) were collected using IQ-TREE to reconstruct the *phnX* phylogeny. Horizontal lines represent the lengths of internal (dark colours) and terminal (pale colours) branches on which gene duplications, transfers and losses are predicted to have occurred.

SUPPLEMENTARY TABLES

Table 1: Phosphorus-cycling genes analysed in this study. Number of homologs is presented alongside the number of genomes which encode each gene and the number of speciations (spe), horizontal gene transfers (hgt), duplications (dup) and losses (los) predicted by ecceTERA using CIR, LN, and UGAM clock models with default event costs.

Phosphorus-Cycling Gene	Homologs	Genomes	CIR				LN				UGAM			
			spe	hgt	dup	los	spe	hgt	dup	los	spe	hgt	dup	los
phnJ	45	45	28	32	0	18	31	31	0	19	27	38	1	21
phnM	69	53	29	64	4	27	29	64	3	27	39	63	5	37
phnZ	42	40	39	29	2	26	38	35	2	29	38	35	2	28
phnX	29	28	9	33	1	10	9	33	1	10	9	32	2	12
phnA	12	12	0	11	0	0	0	11	0	0	0	11	0	0
phnW	28	25	8	28	2	8	8	28	2	8	8	28	2	8
ppd	36	33	11	33	3	11	11	33	3	11	11	34	4	13
pepM	42	41	14	47	0	18	14	47	1	18	14	42	0	15
ptxD	50	49	14	50	3	12	22	52	2	22	22	52	2	23
ptxB	41	38	22	41	0	19	18	39	1	17	18	39	1	17
htxB	5	4	2	3	1	2	2	3	1	2	2	3	1	2
htxA	20	17	10	19	0	11	3	18	3	5	3	18	2	5
pitH	0	0	n/a	n/a	n/a	n/a	n/a	n/a	n/a	n/a	n/a	n/a	n/a	n/a
pitA	1	1	n/a	n/a	n/a	n/a	n/a	n/a	n/a	n/a	n/a	n/a	n/a	n/a
pstS	469	174	93	210	269	84	91	212	268	83	87	212	273	82
pnas	345	257	171	325	33	163	176	329	34	176	176	347	30	186

Table 2: Firmicutes and Deltaproteobacteria diverged from one another in the Paleo- or Eo-archean. Estimated divergence times for the MRCA of Firmicutes and Deltaproteobacteria in molecular clocks constructed with three different models are presented. Ga, billion years ago.

CIR	LN	UGAM
3.45 Ga (3.30 to 3.59)	3.78 Ga (3.90 to 3.66)	3.85 Ga (4.21 to 3.54)

Table 3: The ribosomal proteins used here to reconstruct the tree of life and molecular clock have been utilised in several independent studies for a similar purpose. A complete representation of papers reporting on the topology of the tree of life, including Bacteria and Archaea is included alongside the presence or absence of the ribosomal proteins used in these analyses in their multi-gene datasets. The total number of proteins used in each dataset is listed on the right alongside a column detailing whether they also ran a molecular clock using the same proteins.

	RpL2	RpL3	RpL4	RpL5	RpL6	RpL14	RpL15e	RpL16	RpL18	RpL22	RpL24	RpS3	RpS8	RpS10	RpS17	RpS19	Number of Proteins	Molecular Clock Proteins
[16]	y	y	y	y	y	y	y	y	y	y	n	y	y	n	y	y	40	n/a
[17]	?	?	?	?	?	?	?	?	?	?	?	?	?	?	?	?	400	n/a
[10]	y	y	y	y	y	y	y	y	y	y	y	y	y	y	y	y	16	n/a
[9]	y	y	y	y	y	y	y	n	y	y	y	y	y	n	y	y	14	n/a
[18]	y	n	n	n	n	n	n	n	n	n	n	y	n	n	n	n	29	same
[19]	y	n	n	y	n	n	n	n	n	n	n	y	n	n	n	n	381	same
[14]	y	y	y	y	y	y	y	y	y	y	y	y	y	y	y	y	16	same
[20]	n	n	n	n	n	n	n	n	n	n	n	y	n	n	n	n	21	n/a
[21]	y	y	y	y	y	y	y	y	y	y	y	y	y	y	y	y	16	same
[11]	y	y	y	y	y	y	y	y	y	y	n	y	y	n	y	y	30	n/a
[8]	y	y	n	y	n	y	n	n	n	y	n	y	y	y	n	n	27	same
[22]	y	y	y	y	y	y	y	y	y	y	y	y	y	y	y	y	16	same
[23]	y	y	y	y	y	y	y	y	y	y	n	y	y	n	y	y	30	same

Table 4: Calibration points used in molecular clock analyses. We stress that these are conservative estimates, as the origin of a metabolism or group of organisms may predate its first widely-accepted expression in the rock record.

Calibration	Minimum Age	Citation(s)	Maximum Age	Citation (s)	Phylogenetic Placement	Citation(s)
Methanogenesis	2.7 Ga	[24]	n/a	n/a	MRCA of TACK and Euryarchaeota	[4,5,25]
Red algae	1.05 Ga	[26]	n/a	n/a	MRCA of red algae and red algal chloroplasts	[27]
Oxygenic photosynthesis	2.32 Ga	[28]	2.7 Ga	[29]	MRCA of photosynthetic cyanobacteria	Cyanobacteria were the first producers of biogenic oxygen
Eukaryotes	1.7 Ga	[30]	n/a	n/a	MRCA of Eukaryotes and Archaea	n/a
Akinetes	1.6 Ga	[31]	n/a	n/a	First radiation of heterocyst-forming cyanobacteria	[32]
Prymnesiophyte endosymbionts	91 Ma	[33]	n/a	n/a	MRCA of UCYNA	[33]
Hemiaulus endosymbionts	110 Ma	[34]	n/a	n/a	MRCA of *Richelia intracellularis HH01* and *HM01*	[35]

MRCA: Most recent common ancestor

Table 5: Source of HMM profiles used to identify phosphonate cycling genes. Each was downloaded from an equivalog HMM used in the NCBI's prokaryotic genome annotation pipeline.

Protein	Equivalog HMM
PhnZ	TIGR03276.1
PhnX	TIGR01422.2
PhnA	TIGR02335.1
PhnM	TIGR02318.1
PhnJ	PF06007
PhnW	TIGR02326.1
PepM	TIGR02320.1
Ppd	TIGR03405.1
PstS	TIGR00975.1 & NF008171.0
PNaS	NF037997.1
PitH	NBR010556
PitA	NF03774.1

Table 6: Proteins used to build HMM profiles for PtxD, PtxB, HtxB and HtxA

Protein	NCBI ID	Citation	Organism
PtxD	YP_001091477.1	[36]	*Prochlorococcus marinus str.* MIT 9301
	ADB92513.1	[37]	*Desulfotignum phospitoxidans*
	K18916*	[38]	*Pseudomonas stutzeri* WM88
	AAT12779.1	[39]	*Alcaligenes faecalis* WM2072
PtxB	AAC71707.1	[36,40,41]	*Pseudomonas stutzeri* WM88
	YP_001091475.1	[36,41,42]	*Prochlorococcus marinus str.* MIT 9301
	ABG49835	[41]	*Trichodesmium erythreaum* IMS101
HtxB	AAC71712.1	[41,43]	*Pseudomonas stutzeri* WM88
	AAT12776.1	[39]	*Alcaligenes faecalis* WM2072
HtxA	AAC71711.1	[39,40,44]	*Alcaligenes faecalis* WM2072

* KEGG ID

REFERENCES

1 David, L. A. & Alm, E. J. Rapid evolutionary innovation during an Archaean genetic expansion. *Nature* **469**, 93-96 (2011). https://doi.org:10.1038/nature09649

2 Martinez-Gutierrez, C. A. & Aylward, F. O. Genome size distributions in bacteria and archaea are strongly linked to evolutionary history at broad phylogenetic scales. *Plos Genet.* **18**, e1010220 (2022). https://doi.org:10.1371/journal.pgen.1010220

3 Rodríguez-Gijón, A. *et al.* A genomic perspective across earth's microbiomes reveals that genome size in archaea and bacteria is linked to ecosystem type and trophic strategy. *Front. Microbiol.* **12**, 761869 (2021). https://doi.org:10.3389/fmicb.2021.761869

4 Wang, Y. *et al.* A methylotrophic origin of methanogenesis and early divergence of anaerobic multicarbon alkane metabolism. *Sci. Adv.* **7** (2021). https://doi.org:10.1126/sciadv.abj1453

5 Williams, T. A. *et al.* Integrative modeling of gene and genome evolution roots the archaeal tree of life. *Proc. Natl. Acad. Sci. USA* **114**, E4602-E4611 (2017). https://doi.org:10.1073/pnas.1618463114

6 Witwinowski, J. *et al.* An ancient divide in outer membrane tethering systems in bacteria suggests a mechanism for the diderm-to-monoderm transition. *Nat. Microbiol.* **7**, 411-422 (2022). https://doi.org:10.1038/s41564-022-01066-3

7 Coleman, G. A. *et al.* A rooted phylogeny resolves early bacterial evolution. *Science* **372** (2021). https://doi.org:10.1126/science.abe0511

8 Moody, E. R. R. *et al.* An estimate of the deepest branches of the tree of life from ancient vertically evolving genes. *Elife* **11** (2022). https://doi.org:10.7554/eLife.66695

9 Castelle, C. J. & Banfield, J. F. Major new microbial groups expand diversity and alter our understanding of the tree of life. *Cell* **172**, 1181-1197 (2018). https://doi.org:https://doi.org/10.1016/j.cell.2018.02.016

10 Hug, L. A. *et al.* A new view of the tree of life. *Nat. Microbiol.* **1** (2016). https://doi.org:10.1038/Nmicrobiol.2016.48

11 Martinez-Gutierrez, C. A. & Aylward, F. O. Phylogenetic signal, congruence, and uncertainty across Bacteria and Archaea. *Mol. Biol. Evol.* **38**, 5514-5527 (2021). https://doi.org:10.1093/molbev/msab254

12 Parks, D. H. *et al.* Recovery of nearly 8,000 metagenome-assembled genomes substantially expands the tree of life. *Nat Microbiol* **2**, 1533-1542 (2017). https://doi.org:10.1038/s41564-017-0012-7

13 Taib, N. *et al.* Genome-wide analysis of the Firmicutes illuminates the diderm/monoderm transition. *Nat. Ecol. Evol.* **4**, 1661-1672 (2020). https://doi.org:10.1038/s41559-020-01299-7

14 Chen, S.-C. *et al.* The Great Oxidation Event expanded the genetic repertoire of arsenic metabolism and cycling. *Proc. Natl. Acad. Sci. USA* **117**, 10414-10421 (2020). https://doi.org:doi:10.1073/pnas.2001063117

15 Schulz, F. *et al.* Towards a balanced view of the bacterial tree of life. *Microbiome* **5**, 140 (2017). https://doi.org:10.1186/s40168-017-0360-9

16 Sunagawa, S. *et al.* Metagenomic species profiling using universal phylogenetic marker genes. *Nat. Methods* **10**, 1196-+ (2013). https://doi.org:10.1038/Nmeth.2693

17 Segata, N., Börnigen, D., Morgan, X. C. & Huttenhower, C. PhyloPhlAn is a new method for improved phylogenetic and taxonomic placement of microbes. *Nat. Commun.* **4**, 2304 (2013). https://doi.org:10.1038/ncomms3304

18 Betts, H. C. *et al.* Integrated genomic and fossil evidence illuminates life's early evolution and eukaryote origin. *Nat. Ecol. Evol.* **2**, 1556-1562 (2018). https://doi.org:10.1038/s41559-018-0644-x
19 Zhu, Q. *et al.* Phylogenomics of 10,575 genomes reveals evolutionary proximity between domains Bacteria and Archaea. *Nat. Commun.* **10**, 5477 (2019). https://doi.org:10.1038/s41467-019-13443-4
20 Williams, T. A., Cox, C. J., Foster, P. G., Szollosi, G. J. & Embley, T. M. Phylogenomics provides robust support for a two-domains tree of life. *Nat. Ecol. Evol.* **4**, 138-147 (2020). https://doi.org:10.1038/s41559-019-1040-x
21 Parsons, C., Stueken, E. E., Rosen, C. J., Mateos, K. & Anderson, R. E. Radiation of nitrogen-metabolizing enzymes across the tree of life tracks environmental transitions in Earth history. *Geobiology* **19**, 18-34 (2021). https://doi.org:10.1111/gbi.12419
22 Mateos, K. *et al.* The evolution and spread of sulfur cycling enzymes reflect the redox state of the early Earth. *Sci. Adv.* **9**, eade4847 (2023). https://doi.org:10.1126/sciadv.ade4847
23 Martinez-Gutierrez, C. A., Uyeda, J. C. & Aylward, F. O. A timeline of bacterial and archaeal diversification in the ocean. *Elife* **12**, RP88268 (2023). https://doi.org:10.7554/eLife.88268
24 Eigenbrode, J. L. & Freeman, K. H. Late Archean rise of aerobic microbial ecosystems. *Proc. Natl. Acad. Sci. USA* **103**, 15759-15764 (2006). https://doi.org:10.1073/pnas.0607540103
25 Moore, E. K., Jelen, B. I., Giovannelli, D., Raanan, H. & Falkowski, P. G. Metal availability and the expanding network of microbial metabolisms in the Archaean eon. *Nat. Geosci.* **10**, 629-636 (2017). https://doi.org:10.1038/Ngeo3006
26 Gibson, T. M. *et al.* Precise age of Bangiomorpha pubescens dates the origin of eukaryotic photosynthesis. *Geology* **46**, 135-138 (2018). https://doi.org:10.1130/G39829.1
27 Sanchez-Baracaldo, P., Raven, J. A., Pisani, D. & Knoll, A. H. Early photosynthetic eukaryotes inhabited low-salinity habitats. *Proc. Natl. Acad. Sci. USA* **114**, E7737-E7745 (2017). https://doi.org:10.1073/pnas.1620089114
28 Bekker, A. *et al.* Dating the rise of atmospheric oxygen. *Geochim. Cosmochim. Ac.* **68**, A780-A780 (2004).
29 Bosak, T., Liang, B., Sim, M. S. & Petroff, A. P. Morphological record of oxygenic photosynthesis in conical stromatolites. *Proc. Natl. Acad. Sci. USA* **106**, 10939-10943 (2009). https://doi.org:10.1073/pnas.0900885106
30 Pang, K. *et al.* The nature and origin of nucleus-like intracellular inclusions in Paleoproterozoic eukaryote microfossils. *Geobiology* **11**, 499-510 (2013). https://doi.org:10.1111/gbi.12053
31 Golubic, S., Sergeev, V. N. & Knoll, A. H. Mesoproterozoic Archaeoellipsoides: Akinetes of heterocystous cyanobacteria. *Lethaia* **28**, 285-298 (1995). https://doi.org:10.1111/j.1502-3931.1995.tb01817.x
32 Sanchez-Baracaldo, P., Hayes, P. K. & Blank, C. E. Morphological and habitat evolution in the Cyanobacteria using a compartmentalization approach. *Geobiology* **3**, 145-165 (2005). https://doi.org:10.1111/j.1472-4669.2005.00050.x
33 Cornejo-Castillo, F. M. *et al.* Cyanobacterial symbionts diverged in the late Cretaceous towards lineage-specific nitrogen fixation factories in single-celled phytoplankton. *Nat. Commun.* **7**, 11071 (2016). https://doi.org:10.1038/ncomms11071
34 Sims, A. P., Mann, D. G. & Medlin, L. K. Evolution of the diatoms: Insights from fossil, biological and molecular data. *Phycologia* **45**, 361-402 (2006).

35 Caputo, A., Stenegren, M., Pernice, M. C. & Foster, R. A. A short comparison of two marine planktonic diazotrophic symbioses highlights an un-quantified disparity. *Front. Mar. Sci.* **5** (2018). https://doi.org:10.3389/fmars.2018.00002

36 Martinez, A., Osburne, M. S., Sharma, A. K., DeLong, E. F. & Chisholm, S. W. Phosphite utilization by the marine picocyanobacterium *Prochlorococcus* MIT9301. *Environ. Microbiol.* **14**, 1363-1377 (2012). https://doi.org:10.1111/j.1462-2920.2011.02612.x

37 Simeonova, D. D., Wilson, M. M., Metcalf, W. W. & Schink, B. Identification and heterologous expression of genes involved in anaerobic dissimilatory phosphite oxidation by *Desulfotignum phosphitoxidans*. *J. Bacteriol.* **192**, 5237-5244 (2010). https://doi.org:10.1128/JB.00541-10

38 Costas, A. M. G., White, A. K. & Metcalf, W. W. Purification and characterization of a novel phosphorus-oxidizing enzyme from *Pseudomonas stutzeri* WM88*210. *J. Biol. Chem.* **276**, 17429-17436 (2001). https://doi.org:https://doi.org/10.1074/jbc.M011764200

39 Wilson, M. M. & Metcalf, W. W. Genetic diversity and horizontal transfer of genes involved in oxidation of reduced phosphorus compounds by *Alcaligenes faecalis* WM2072. *Appl. Environ. Microb.* **71**, 290-296 (2005). https://doi.org:10.1128/AEM.71.1.290-296.2005

40 Metcalf, W. W. & Wolfe, R. S. Molecular genetic analysis of phosphite and hypophosphite oxidation by *Pseudomonas stutzeri* WM88. *J. Bacteriol.* **180**, 5547-5558 (1998). https://doi.org:10.1128/JB.180.21.5547-5558.1998

41 Bisson, C. *et al.* The molecular basis of phosphite and hypophosphite recognition by ABC-transporters. *Nat. Commun.* **8**, 1746 (2017). https://doi.org:10.1038/s41467-017-01226-8

42 Feingersch, R. *et al.* Potential for phosphite and phosphonate utilization by Prochlorococcus. *ISME J.* **6**, 827-834 (2012). https://doi.org:10.1038/ismej.2011.149

43 Adams, N. B. P., Robertson, A. J., Hunter, C. N., Hitchcock, A. & Bisson, C. Phosphite binding by the HtxB periplasmic binding protein depends on the protonation state of the ligand. *Sci. Rep.* **9**, 10231 (2019). https://doi.org:10.1038/s41598-019-46557-2

44 White, A. K. & Metcalf, W. W. Isolation and biochemical characterization of hypophosphite/2-oxoglutarate dioxygenase. A novel phosphorus-oxidizing enzyme from *Psuedomonas stutzeri* WM88. *J. Biol. Chem.* **277**, 38262-38271 (2002). https://doi.org:10.1074/jbc.M204605200
