# Supplementary material for: Timing the evolution of phosphorus-cycling enzymes through geological time using phylogenomics
Source: Nat Commun. 2024 May 2;15:3703. doi: 10.1038/s41467-024-47914-0 (PMC11066067; doi:10.1038/s41467-024-47914-0)
Supplement: Supplementary file 3 — Description of Additional Supplementary Files [file 41467_2024_47914_MOESM3_ESM.pdf]

Description of Additional Supplementary Files

Supplementary Data 1

Description: Accession numbers of genomes used in the manuscript
