# Supplementary material for: Timing the evolution of phosphorus-cycling enzymes through geological time using phylogenomics
Source: Nat Commun. 2024 May 2;15:3703. doi: 10.1038/s41467-024-47914-0 (PMC11066067; doi:10.1038/s41467-024-47914-0)
Supplement: Supplementary file 5 — Reporting Summary [file 41467_2024_47914_MOESM5_ESM.pdf]

nature portfolio

Corresponding author(s): Joanne Boden

Last updated by author(s): 13/3/24

Reporting Summary

Nature Portfolio wishes to improve the reproducibility of the work that we publish. This form provides structure for consistency and transparency in reporting. For further information on Nature Portfolio policies, see our Editorial Policies and the Editorial Policy Checklist.

Statistics

For all statistical analyses, confirm that the following items are present in the figure legend, table legend, main text, or Methods section.

n/a	Confirmed	
☐	☒	The exact sample size (*n*) for each experimental group/condition, given as a discrete number and unit of measurement
☒	☐	A statement on whether measurements were taken from distinct samples or whether the same sample was measured repeatedly
☐	☒	The statistical test(s) used AND whether they are one- or two-sided<br>*Only common tests should be described solely by name; describe more complex techniques in the Methods section.*
☒	☐	A description of all covariates tested
☒	☐	A description of any assumptions or corrections, such as tests of normality and adjustment for multiple comparisons
☐	☒	A full description of the statistical parameters including central tendency (e.g. means) or other basic estimates (e.g. regression coefficient) AND variation (e.g. standard deviation) or associated estimates of uncertainty (e.g. confidence intervals)
☐	☒	For null hypothesis testing, the test statistic (e.g. *F*, *t*, *r*) with confidence intervals, effect sizes, degrees of freedom and *P* value noted<br>*Give P values as exact values whenever suitable.*
☐	☒	For Bayesian analysis, information on the choice of priors and Markov chain Monte Carlo settings
☒	☐	For hierarchical and complex designs, identification of the appropriate level for tests and full reporting of outcomes
☒	☐	Estimates of effect sizes (e.g. Cohen's *d*, Pearson's *r*), indicating how they were calculated

Our web collection on statistics for biologists contains articles on many of the points above.

Software and code

Policy information about availability of computer code

Data collection	Outputs of gene-tree-species-tree reconciliations were parsed using python scripts first published in Mateos et al, 2023 and amended to report only the right node of speciation events.
Data analysis	Data were analysed with the following open-source software: HMMER3 v.3.3.2 (homolog collection), MUSCLE v.5.1 and MAFFT v.7.4 (sequence alignment), IQTREE v.2.0.3 and MrBayes v.3.2.7a (tree reconstruction), Phylobayes v.4.1 (bayeisan molecular clocks), R v.4.2.2 (regression analyses and graphs), Python v. 2.7.5 (parsing recPhyloXML files), ecceTERA v.1.2.5 (gene-tree-species-tree reconciliation), trimAl v1.4rev15 (alignment trimming), GToTree v1.6.34 (collection and alignment of ribosomal proteins for species tree), AliView v.1.28 (viewing alignments), TreeViewer v2.0.1, FigTree v1.4.4 and iTOL v6.8 (viewing and annotating evolutionary trees).

For manuscripts utilizing custom algorithms or software that are central to the research but not yet described in published literature, software must be made available to editors and reviewers. We strongly encourage code deposition in a community repository (e.g. GitHub). See the Nature Portfolio guidelines for submitting code & software for further information.

Data

Policy information about availability of data

All manuscripts must include a data availability statement. This statement should provide the following information, where applicable:

- Accession codes, unique identifiers, or web links for publicly available datasets
- A description of any restrictions on data availability
- For clinical datasets or third party data, please ensure that the statement adheres to our policy

TheAll alignments data, protein phylogenies, molecular clocks and the species tree generated in this study are available in the open science framework repository, https://osf.io/vt5rw/?view_only=b13a53f4d87c44d1a82a18b176523c5b. Source data for Figures 3 and 4 and Supplementary Figures 1-16 are provided with this paper in this same repository. Accession numbers of all genomes analysed in this study are provided in the Supplementary Data and accession numbers of HMM profiles and query sequences are provided in the supplementary tables 5 and 6.

Research involving human participants, their data, or biological material

Policy information about studies with human participants or human data. See also policy information about sex, gender (identity/presentation), and sexual orientation and race, ethnicity and racism.

Reporting on sex and gender	n/a
Reporting on race, ethnicity, or other socially relevant groupings	n/a
Population characteristics	n/a
Recruitment	n/a
Ethics oversight	n/a

Note that full information on the approval of the study protocol must also be provided in the manuscript.

Field-specific reporting

Please select the one below that is the best fit for your research. If you are not sure, read the appropriate sections before making your selection.

☒ Life sciences ☐ Behavioural & social sciences ☐ Ecological, evolutionary & environmental sciences

For a reference copy of the document with all sections, see nature.com/documents/nr-reporting-summary-flat.pdf

Life sciences study design

All studies must disclose on these points even when the disclosure is negative.

Sample size	We analysed 865 genomes, including at least one from each order of the tree of life using information from the Genome Taxonomy Database release 95. This method was chosen to enable as large-as-possible representation of the diversity of life on Earth, whilst still maintaining a small enough sample size for molecular clocks to converge in a reasonable amount of time. Where possible, a representative with the gene ptxD was included to better capture the diversity of phosphite-oxidising organisms.
Data exclusions	Homologs were collected using HMMER, and all hits with e values less than 0.1 were excluded. Further hits were excluded based on their phylogenetic relationship to experimentally-identified query sequences (further details in manuscript). Positions in alignments of phosphorus-cycling genes that contained gaps in 70% of sequenced were removed to ensure phylogenies were reconstructed using the most reliable information.
Replication	Each bayesian molecular clock was replicated three times, and the results compared to estimate convergence. Each bayesian gene tree was replicated twice and the results compared to estimate convergence.
Randomization	No experimental set-ups with controls were used, so randomization was not necessary
Blinding	No experimental set-ups with controls were used, so blinding was not necessary

Reporting for specific materials, systems and methods

We require information from authors about some types of materials, experimental systems and methods used in many studies. Here, indicate whether each material, system or method listed is relevant to your study. If you are not sure if a list item applies to your research, read the appropriate section before selecting a response.

Materials & experimental systems

n/a	Involved in the study
☒	☐ Antibodies
☒	☐ Eukaryotic cell lines
☒	☐ Palaeontology and archaeology
☒	☐ Animals and other organisms
☒	☐ Clinical data
☒	☐ Dual use research of concern
☒	☐ Plants

Methods

n/a	Involved in the study
☒	☐ ChIP-seq
☒	☐ Flow cytometry
☒	☐ MRI-based neuroimaging
